# Ferritin light-chain contributes to hair cells function and survival

**DOI:** 10.1038/s42003-026-10069-3

**Published:** 2026-04-18

**Authors:** Chloé P. Petit, Sahia Mahaman Bachir Dodo, Lina María Jaime Tobón, Anne-Gabrielle Harrus, Cécilia Souyris, Antoine Picot, Jing Wang, Rémy Pujol, Frédéric Venail, Ruben Vidal, Jérôme Bourien, Jean-Luc Puel, Régis Nouvian

**Affiliations:** 1 INM, Univ Montpellier, Inserm, Montpellier, France; 2 INM, Univ Montpellier, Inserm, CHU Montpellier, Montpellier, France; 3 Stark Neurosciences Research Institute, Indiana University School of Medicine, Indianapolis, USA; 4 INM, Univ Montpellier, Inserm, CNRS, Montpellier, France

**Keywords:** Cochlea, Hair cell

## Abstract

Iron is essential for cellular function, as it is required for oxygen transport and mitochondria oxidative respiration. However, uncoordinated regulation of intracellular iron can produce reactive oxygen species, calling for a tight homeostasis. Here, we examine the role of the iron-binding protein, ferritin, known to store intracellular iron, in the cochlea. Genetic ablation of the ferritin light-chain (*Ftl*) in mouse leads to two different phenotypes. A fraction of the homozygous mice has moderate to profound hearing loss (*Ftl*^−*/*−^ with High-Threshold, HT), with the other fraction of mice unaffected, i.e., with normal auditory threshold (*Ftl*^−*/*−^ with Low-Threshold, LT). In the *Ftl*^−*/*−^ *HT* mice, the outer hair cells, which amplify incoming sound-stimulation, undergo a massive degeneration and the inner hair cells, which converts the mechanical pressure into exocytosis of glutamate, harbor splayed hair bundles. In addition, patch-clamp recordings demonstrated the alteration of calcium current in the IHCs from *Ftl*^−*/*−^ *HT* mice. Finally, the *Ftl*^−*/*−^ *LT* mice were found to be more vulnerable to acoustic exposure, suggesting that the difference between the two phenotypes may partly stem from noxious environment. Taken together, our results suggest that ferritin light-chain is a contributing factor to the hair cell function and survival.

## Abstract (web summary)

Deletion of the light-chain of ferritin sheds light on the role of iron homeostasis in survival and function of hair cells of the cochlea in a mouse model.

## Introduction

Iron is a crucial element for cell physiology given its role in oxygen transport, electron transfer reactions as well as in gene expression^1,2^. However, its ability to change its oxidation state makes iron highly harmful for cellular organisms calling for a tight homeostatic regulation^3–5^. Among the proteins governing iron metabolism, ferritin is known to sequester and store intracellular iron. Ferritin is composed by two sub-units, the heavy- and light-chain, that assemble into a 24-subunit polymer^6^. While the heavy chain (Fth) has a ferroxidase activity, converting the ferrous iron into ferric iron, the light-chain (Ftl) stabilizes the ferritin shell-like structure and maintain the iron storage^7^. Thus, ferritin has a pivotal role in preventing the formation of radical’s hydroxyls through the Fenton reaction. Although ferritin’s role has been demonstrated in several organs including heart, liver and brain^8–10^, its function in the cochlea remains unknown.

The auditory system is able to encode acoustic cues for a long period of time without noticeable fatigue with frequency up to several ten of kilohertz through a broad range of intensities. Therefore, the encoding activity of the cochlea should set high demands on the cellular metabolism. The cochlea relies on two kinds of sensory cells: the outer hair cells (OHCs) and inner hair cells (IHCs). To detect the incoming acoustic cues, both hair cells harbor a stereocilia bundle, in which the mechanotransducer channels are located^11,12^. The deflection of the stereocilia evoked by sound stimulation leads to the cation influx through the mechanotransducer channel and to the hair cell’s depolarization. Whereas the OHC amplifies the input stimulation and is responsible for exquisite frequency selectivity, depolarization of the IHC opens the voltage-gated calcium channels and the calcium influx triggers the release of the synaptic vesicles filled with glutamate onto the afferent auditory nerve terminals^13,14^. The neural message, i.e., train of action potentials, is then conveyed through the auditory nerve fibers to the cochlear nuclei^15^. To determine the role of ferritin in the cochlea, we took advantage of the knock-out mice for the ferritin-light chain^16^. We found that in a subset of the transgenic mice investigated, deletion of Ftl altered hair cell activity and survival, suggesting that iron homeostasis is critical for auditory function.

## Results

### Hearing loss in a fraction of *Ftl*^−*/*−^ mice

**Fig. 1:**
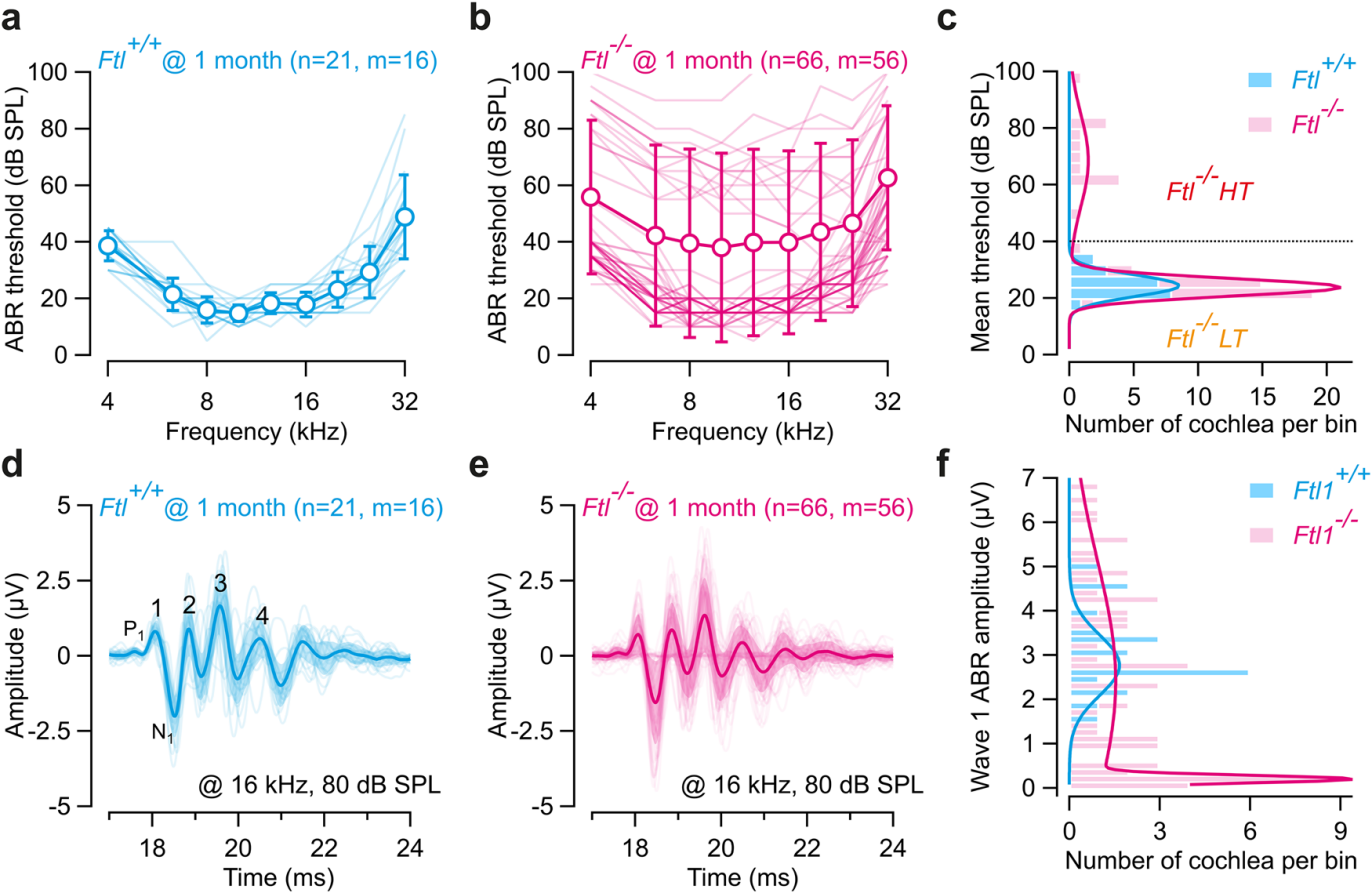
Ferritin light chain knock-out (*Ftl*^−*/*−^) mice show moderate to profound hearing loss. Audiograms (**a**, **b**) and ABR evoked by a 16 kHz tone burst at 80 dB SPL (**d**, **e**) in 1-month-old wild-type mice (*Ftl*^*+/+*^, blue, **a**, **d**) and ferritin light chain knock-out mice (*Ftl*^−*/*−^, magenta, **b**, **e**). Thin lines correspond to individual recordings. Thick lines indicate the mean ± SD. n and m indicate the number of recorded cochlea and mice, respectively. For *Ftl*^*+/+*^ mice, m = 10 males and 11 females. For *Ftl*^−*/*−^ mice, m = 29 males and 27 females. In (**d**), numbers indicate the waves corresponding to the nuclei activation in the auditory pathway. The amplitude of the first wave is measured between the positive (P_1_) and negative peak (N_1_). **c**, **f** Distribution of mean auditory thresholds (**c**, bin width: 4 dB) and wave 1 ABR amplitudes (**f**, bin width: 0.15 µV) obtained from (**a**, **b**) and (**d**, **e**). In (**c**), the pool of mice with auditory threshold beyond 100 dB SPL are nor represented. The dashed black line segregates the pools of *Ftl*^−*/*−^ mice with high- and low-auditory threshold, denoted by *Ftl*^−*/*−^*HT* mice and *Ftl*^−*/*−^ *LT* mice, respectively. Histograms were fitted with a single or two Gaussian (thick lines) for *Ftl*^*+/+*^ (blue) and *Ftl*^−*/*−^ mice (magenta), respectively.

To examine the role of Ftl in the cochlea, we studied the phenotype of the ferritin light-chain subunit knock-out (*Ftl*^−*/*−^) mouse^16^. We first measured the audiograms of the *Ftl*^−*/*−^ mouse using the auditory brainstem response (ABR) that corresponds to the synchronous activation of the nuclei along the ascending auditory pathway (Fig. 1). Deletion of Ftl in the homozygous 1-month-old mice led to a moderate hearing loss in comparison to the wild-type mice of the same age (mean auditory threshold, *Ftl*^*+/+*^: 25.3 ± 4.3 dB SPL and *Ftl*^−*/*−^: 45.3 ± 30.2 dB SPL; *P* = 0.044, two-tailed Mann-Whitney U test). However, the audiogram waveforms in the *Ftl*^−*/*−^ mice seemed to be widely scattered. While audiograms that looked similar to the wild-type mice were recorded in most of the *Ftl*^−*/*−^ mice, elevated auditory thresholds were measured in a fraction of homozygous mice (Fig. 1a, b). The diversity in the audiograms was reflected through the mean auditory threshold distribution. The mean auditory thresholds of wild-type mice followed a normal distribution (*P* = 0.71, Shapiro-Wilk test) and was well fitted by a Gaussian function. In contrast, the data from homozygous mice did not follow a normal distribution (*P* = 2.10^−9^, Shapiro-Wilk test) but was better fitted by a double Gaussian function (Fig. 1c). These results suggested that the deletion of Ftl led to at least two distinct phenotypes, i.e., *Ftl*^−*/*−^ mice with hearing threshold similar to the wild-type (low-threshold, LT) and *Ftl*^−*/*−^ mice having elevated thresholds (high-threshold, HT).

The different phenotypes in the *Ftl*^−*/*−^ mice were also observed in ABR to pure-tone stimulation. Robust responses were measured in most of the homozygous mice except a small fraction of *Ftl*^−*/*−^ mice, in which ABR amplitudes were reduced or even any waves could be discerned (Fig. 1d, e). Because of the small number of *Ftl*^−*/*−^ mice with decreased ABR, the average of the first ABR wave amplitude did not significantly differ between the genotypes (wave1_amp_ @ 16 kHz, 80 dB SPL, *Ftl*^*+/+*^: 3 ± 0.9 µV; *Ftl*^−*/*−^: 2.5 ± 1.9 µV; *P* = 0.23, two-tailed Mann-Whitney U test). However, akin to the audiograms, the distribution of the first ABR wave amplitude followed a normal distribution (*P* = 0.25, Shapiro-Wilk test) and was well fitted by a Gaussian function, while the data from the homozygous mice did not follow a normal distribution (*P* = 8.10^−4^, Shapiro-Wilk test) but was better fitted by a double Gaussian function (Fig. 1f). Given the variability in the ABR threshold and amplitude among the *Ftl*^−*/*−^ mice, we decided to split the *Ftl*^−*/*−^ mice into two groups according to their mean auditory threshold, i.e, *Ftl*^−*/*−^ mice with low-threshold (*Ftl*^−*/*−^ *LT* mice), in which the mean auditory threshold was below 40 dB SPL, and *Ftl*^−*/*−^ mice with high-threshold (*Ftl*^−*/*−^ *HT* mice) having a mean auditory threshold above 40 dB SPL (Fig. 1c). Based on this criterion, 23% of the homozygous mice were classified as *Ft1*^−*/*−^ *HT* in our first screen (Fig. 1) and this proportion was 18% across the entire study.

**Fig. 2:**
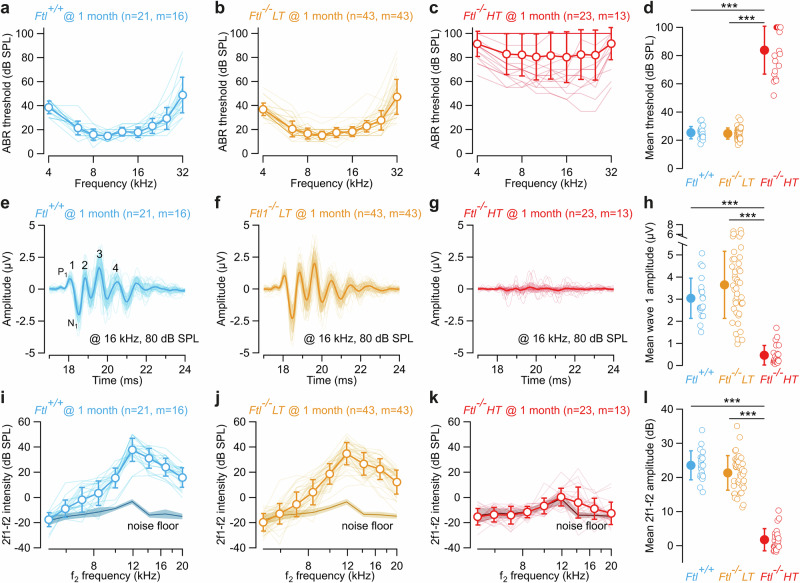
Loss of Ftl leads to profound hearing deficit in a fraction of *Ftl*^−*/*−^ mice. Audiograms (**a–c**), ABR (**e**–**g**) and DPOAEs (**i–k**) in 1-month-old wild-type (*Ftl*^*+/+*^, blue, **a**, **e**, **i**) and ferritin light chain knock-out mice with low auditory thresholds (*Ftl*^−*/*−^ *LT*, orange, **b**, **f**, **j**) and high auditory thresholds (*Ftl*^−*/*−^ *HT*, red, **c**, **g**, **k**). Thin lines indicate individual recordings. Thick lines indicate the mean ± SD. n and m indicate the number of recorded cochlea and mice, respectively. For *Ftl*^*+/+*^ mice, m = 10 males and 11 females. For *Ftl*^−*/*−^ *LT* mice, m = 22 males and 21 females. For *Ftl*^−*/*−^ *HT* mice, m = 7 males and 6 females. In (**e**), numbers indicate the waves corresponding to the nuclei activation in the auditory pathway. The amplitude of the first wave is measured between the positive (P_1_) and negative peak (N_1_). **d**, **h**, **l** Mean auditory thresholds (**d**), wave 1 ABR amplitudes (**h**) and DPOAEs amplitudes (**l**) from *Ftl*^*+/+*^ (blue), *Ftl*^−*/*−^ *LT*, (orange) and *Ftl*^−*/*−^ *HT* mice (red). Bar graphs represent mean ± SD. Circles correspond to individual measurements.

As expected, the mean auditory threshold was significantly elevated in *Ftl*^−*/*−^ *HT* mice (83.8 ± 17 dB SPL) compared to *Ftl*^*+/+*^ and *Ftl*^−*/*−^ *LT* mice (25.3 ± 4.3 dB SPL and 24.7 ± 3.9 dB SPL, respectively; Kruskal-Wallis test *P* < 0.0001; post hoc Dunn’s test: *P* < 0.0001 for *Ftl*^*+/+*^ versus *Ftl*^−*/*−^ *HT* and *P* < 0.0001 for *Ftl*^−*/*−^ *LT* versus *Ftl*^−*/*−^ *HT*; Fig. 2a–d). In contrast, *Ftl*^*+/+*^ and *Ftl*^−*/*−^ *LT* mice had comparable audiograms (post hoc Dunn’s test: *P* > 0.99; Fig. 2a, b, d). In the same manner, smaller ABR amplitude was measured in the *Ftl*^−*/*−^ *HT* mice in comparison to *Ftl*^*+/+*^ wild-type mice and *Ftl*^−*/*−^ *LT* mice (wave1_amp_ @ 16 kHz, 80 dB SPL, *Ftl*^−*/*−^ *HT*: 0.4 ± 0.4 µV versus *Ftl*^*+/+*^: 3 ± 0.9 µV and *Ftl*^−*/*−^ *LT*: 3.6 ± 1.5 µV; Kruskal-Wallis test *P* < 0.0001; post hoc Dunn’s test: *P* < 0.0001 and *P* < 0.0001 in comparison to *Ftl*^*+/+*^ and *Ftl*^−*/*−^ *LT* mice, respectively; Fig. 2e–h). No significant difference in the ABR wave 1 amplitude was found between *Ftl*^*+/+*^ mice and *Ftl*^−*/*−^ *LT* mice (post hoc Dunn’s test: *P* = 0.6597; Fig. 2e, f, h). To know whether the hearing deficit was due to the loss of functional OHCs, we measured the distortion products of the otoacoustic emissions (DPOAEs). In contrast to *Ftl*^*+/+*^ mice and *Ftl*^−*/*−^ *LT* mice, the DPOAEs were reduced in the *Ftl*^−*/*−^ *HT* mice, indicating a deficit in the OHCs mechanical activity (mean 2f_1_−f_2_ @ f_2_ from 5 to 20 kHz: 23.5 ± 4.3 dB, 21.3 ± 5.1 dB and 1.7 ± 3.2 dB in 1-month-old *Ftl*^*+/+*^ mice, *Ftl*^−*/*−^ *LT* mice and *Ftl*^−*/*−^ *HT* mice, respectively; Kruskal-Wallis test *P* < 0.0001; post hoc Dunn’s test: *P* < 0.0001 for *Ftl*^*+/+*^ versus *Ftl*^−*/*−^ *HT, P* < 0.0001 for *Ftl*^−*/*−^ *LT* versus *Ftl*^−*/*−^ *HT* and *P* = 0.6122 for *Ftl*^*+/+*^ versus *Ftl*^−*/*−^ *LT*; Fig. 2i–l). However, a reduction in the active mechanisms would lead to a threshold shift up to 60 dB but cannot account for a complete hearing loss and for the ABR reduction evoked by high level of stimulation as seen in some of the *Ftl*^−*/*−^ *HT* mice^17,18^.

As the mechanotransduction in hair cells strongly depends on the stria vascularis activity^15^, we therefore probed the endocochlear potential, which reflects the functional activity of the stria vascularis. Insertion of a microelectrode in the *Scala media* showed a positive endocochlear potential up to +100 mV in the *Ftl*^*+/+*^ mice. In contrast, we found smaller endocochlear potentials in 4 out of 5 *Ftl*^−*/*−^ HT mice (EP in *Ftl*^*+/+*^: 106.5 ± 6.9 mV, range 98–115 mV, *n* = 4 and in *Ftl*^−*/*−^ *HT*: 49.6 ± 30, range 17–99 mV, *n* = 5; *P* = 0.031, two-tailed Mann-Whitney U test). These results suggested that a functional defect in the stria vascularis contributes to some extent to the hearing loss in the *Ftl*^−*/*−^ HT mice.

### Morphological defects in hair cells from *Ftl*^−*/*−^ mice

**Fig. 3:**
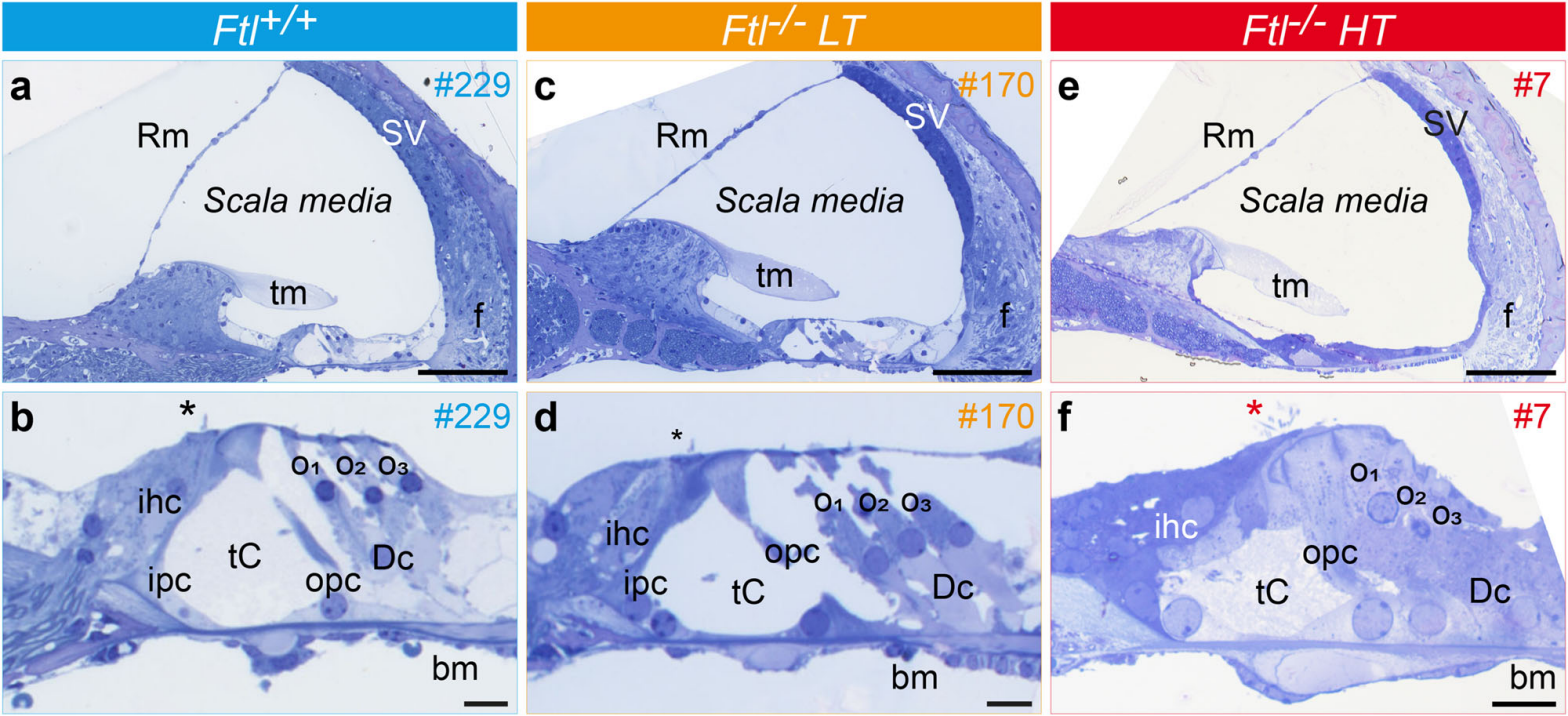
Collapsed organ of Corti in *Ftl*^−*/*−^*HT* mice. **a**, **b** Section of the middle turn from 1-month-old wild-type *Ftl*^*+/+*^ mice cochlea. In (**a**), the stria vascularis (SV), fibrocytes (**f**), tectorial membrane (tm) and Reissner membrane (Rm) had a normal appearance. In (**b**), note all three intact OHCs (o_1_, o_2_ and o_3_), separated from each other and from the outer pillar cells (opc) by large spaces of Nuel. The tunnel of Corti (tC), separated by the inner (ipc) and opc, has a normal appearance as well as the inner hair cells (ihc) with erected stereocilia (black asterisk, **b**). **c**, **d** Section of the middle turn from 1-month-old *Ftl*^−*/*−^ *LT* mice cochlea. The overall organization of the organ of Corti is similar to the wild-type cochlea. **e**, **f** Section of the middle turn from P19 *Ftl*^−*/*−^ *HT* mice cochlea. In (**e**), the stria vascularis (SV), fibrocytes (**f**), tectorial membrane (tm) and Reissner membrane (Rm) had a normal appearance. In (**f**), note the greatly damaged OHCs and the absence of spaces of Nuel. The tunnel of Corti (tC) contains material, that may correspond to cellular debris. Inner hair cells (ihc) seems to have a normal appearance except for disarrayed stereocilia (red asterisk, **f**). bm basilar membrane, Dc Deiter’s cell. Scale bars: (**a**, **c**, **e**): 100 μm; (**b**, **d**, **f**): 10 µm. # indicates the animal’s number.

To determine whether the functional deficit is correlated to any morphological alteration within the cochlea, we carried-out anatomical observations. Light microscopy examinations from semi-thin preparations of *Ftl*^*+/+*^ mice showed normal organs of Corti, with a single row of IHC and three rows of OHCs, separated by the tunnel of Corti (Fig. 3a, b; *n* = 6 cochleae, 6 mice). Consistent with the normal auditory physiology, the structural organization of the cochlea in *Ftl*^−*/*−^ *LT* mice was comparable to the wild-type mice, e.g., IHCs harbored erected stereocilia, OHCs interspaced by the spaces of Nuel and the pillar cells delimitating the tunnel of Corti (Fig. 3c, d; *n* = 3 cochleae, 3 mice). In the *Ftl*^−*/*−^ *HT* mice, the epithelium was disorganized with the loss of the spaces of Nuel and damaged OHCs (Fig. 3e, f*;* *n* = 4 cochleae, 3 mice). The IHCs remained at first glance intact, except for their stereociliary bundle, which appeared splayed (Fig. 3f). The fibrocytes of the outer sulcus and the Reissner membrane seemed well preserved (Fig. 3e). Although we measured a drop in the endocochlear potential, the stria vascularis did not show any sign of degeneration (area of the stria vascularis: *Ftl*^*+/+*^ mice: 5140 ± 751 µm^2^, *n* = 6 cochlea from 6 mice; *Ftl*^−*/*−^ *LT* mice: 6305 ± 1895 µm^2^, *n* = 3 cochlea from 3 mice; *Ftl*^−*/*−^ *HT* mice: 5918 ± 1232 µm^2^, *n* = 4 cochlea from 3 mice; *P* = 0.377, one way ANOVA test).

**Fig. 4:**
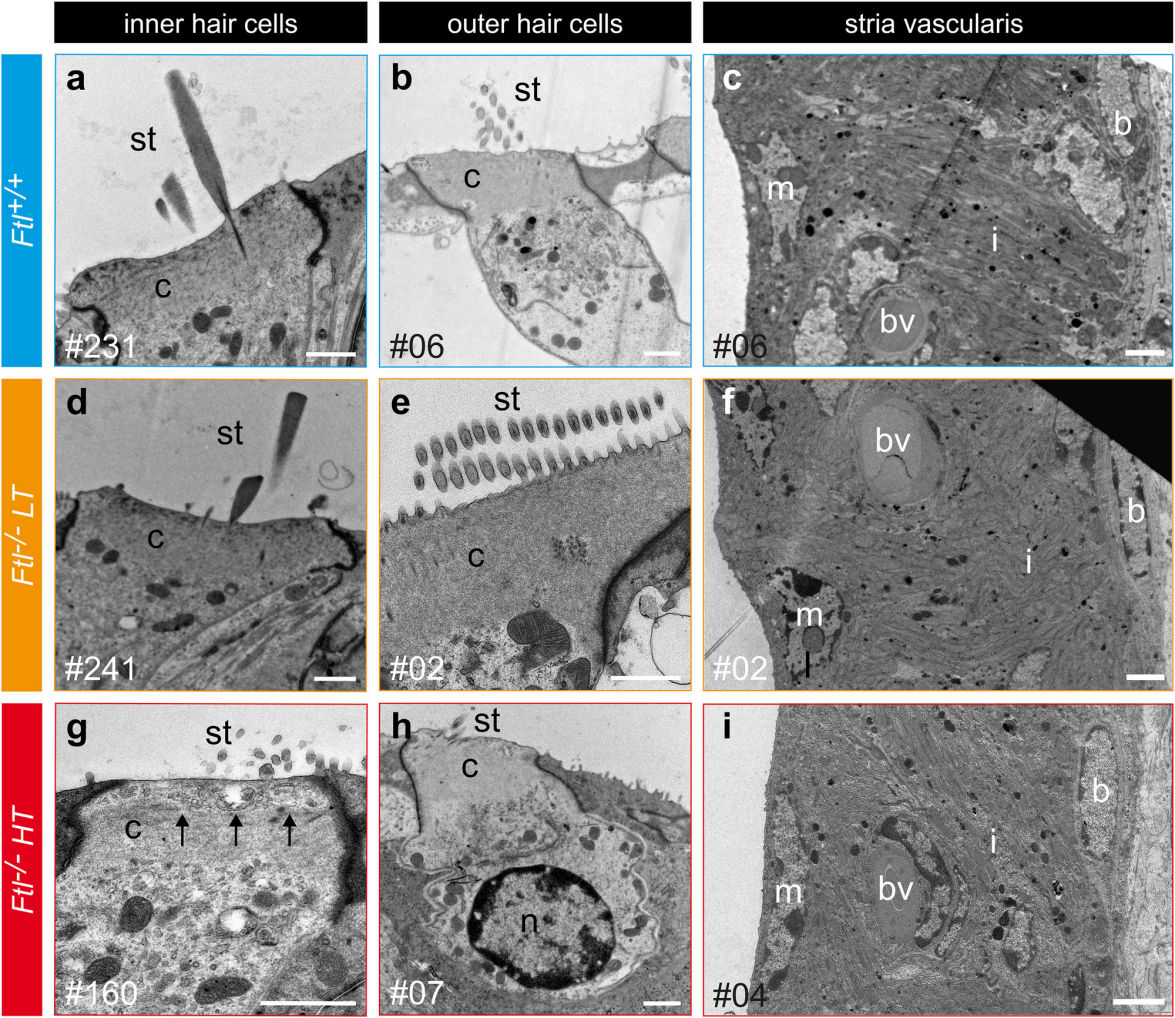
Damage of hair cells in the *Ftl*^−*/*−^*HT* mice. Transmission electron microscopy observations of inner hair cells (**a**: P19, **d**: 1 month, **g**: 1 month), outer hair cells (**b**: P18, E: P18 and H: P19) and stria vascularis epithelium (**c**: P18, **f**: P18 and **i**: P18) from *Ftl*^*+/+*^ mice (**a**–**c**), *Ftl*^−*/*−^ *LT* mice (**d**–**f**) and *Ftl*^−*/*−^ *HT* mice (**g**–**i**). In the *Ftl*^−*/*−^ *HT* mice, note the abnormal presence of numerous vacuoles within the cuticular plate of the IHC (arrows, **g**) and the shrunked OHC (**h**). Also note the chromatin compaction indicating apoptosis in the OHC nucleus. Scale bars: **a**, **b,** **d,** **e,** **g**, **h**: 1 μm; **c**, **f**, **i**: 2 µm. # indicates the animal’s number. st stereocilia bundle, c cuticular plate, n nucleus, m marginal cell, i intermediate cell, b basal cell, bv blood vessel.

In TEM, we observed abnormal vacuoles within the cuticular plate of hair cells in 2 samples out of 4 from *Ftl*^−*/*−^ *HT* mice (Fig. 4g), contrasting the well-implanted stereocilia bundle within the cuticular plate in the *Ftl*^*+/+*^ mice (Fig. 4a, b) and *Ftl*^−*/*−^ *LT* mice (Fig. 4d, e). In all the samples from *Ftl*^−*/*−^ *HT* mice, the cell soma of OHCs was distorted or shrunken within a collapsed epithelium (Fig. 4h). By contrast, the morphology of the three layers of the stria vascularis, i.e., the basal cells, the intermediate cells and the marginal cells, was undistinguishable between wild-type, *Ftl*^−*/*−^ *LT* mice and in *Ftl*^−*/*−^ *HT* mice (Fig. 4c, f, i).

**Fig. 5:**
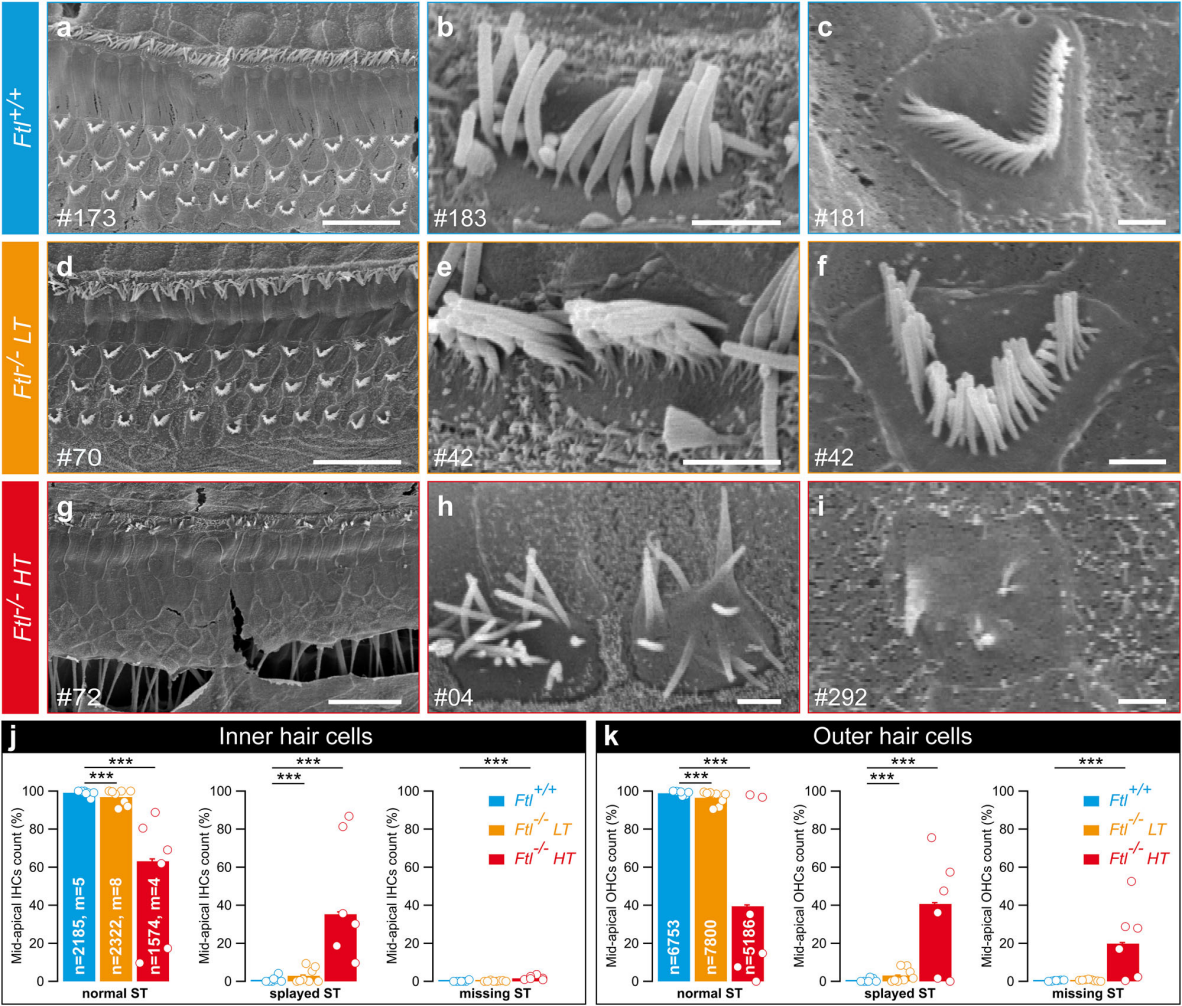
Ultrastructure defect of hair cells from *Ftl*^−*/*−^*HT* mice. Scanning electron microscopy of the organ of Corti (**a**: P30, **d**: P26 and **g**: P26), stereocili bundle from inner (**b**: P19, **e**: P19 and **h**: P18) and outer (**c**: P19, **f**: P19 and **i**: P21) hair cells at mid-apical turns from *Ftl*^*+/+*^ mice, *Ftl*^−*/*−^ *LT* mice and *Ftl*^−*/*−^ *HT* mice. # indicates the animal’s number. IHCs inner hair cells, OHCs: outer hair cells. Scale bars: 15 μm in (**a**, **d**, **g)** and 2 μm in (**b**, **e**, **h**, **c**, **f**, **i**). **j**, **k** Percentages of inner (**j**) and outer (**k**) hair cells harboring normal, splayed or missing stereocilia (ST). Bar plots correspond to mean ± SD. Circles represent individual cochleas. n and m indicate the number of hair cells examined and mice, respectively.

To quantify the number of damaged hair cells, we used SEM. The apical side of most of the hair cells in the *Ftl*^*+/+*^ mice projected well-organized stereociliary bundle (Fig. 5a–c; *n* = 7 cochleae, 5 mice). The fraction of IHCs with altered stereocilia was small in the *Ftl*^−*/*−^ *LT* mice (3%, Fig. 5d, e; *n* = 8 cochleae, 8 mice), but reached a significant difference in comparison to wild-type mice (0.7% of IHCs with splayed stereocilia; *P* = 1.10^−7^, 2-sample test for equality of proportions with continuity correction; Fig. 5j). In *Ftl*^−*/*−^ *HT* mice, more than one-third (35%) of IHCs harbored distorted or fused stereocilia (*P* = 2.10^−16^ in comparison to *Ftl*^*+/+*^ mice; 2-sample test for equality of proportions with continuity correction; *n* = 6 cochleae, 4 mice; Fig. 5g, h, j). Only a small number of IHCs without stereocilia were found in the *Ftl*^−*/*−^ *HT* mice (1.6% against 0.09% in *Ftl*^*+/+*^ mice, *P* = 2.10^−7^, 2-sample test for equality of proportions with continuity correction; Fig. 5g, j).

In the same manner, the number of OHCs with abnormal stereocilia architecture was sparse but significantly larger in *Ftl*^−*/*−^ *LT* mice (3%) compared to *Ftl*^*+/+*^ mice (0.7%; *P* = 2.10^−16^, 2-sample test for equality of proportions with continuity correction; Fig. 5f, k). By contrast, 40% of the OHCs harbored splayed stereocilia bundle in the *Ftl*^−*/*−^ *HT* mice (*P* = 2.10^−16^ in comparison to wild-type mice, 2-sample test for equality of proportions with continuity correction; Fig. 5I, k). In addition, nearly 20% of the OHCs were found without stereocilia (*P* = 2.10^−16^ in comparison to wild-type mice, 2-sample test for equality of proportions with continuity correction; Fig. 5g, k). Although the degree of hair cell damage could vary, e.g., ranging from epithelium with almost entire population of OHCs with normal stereocilia (2 cochleae out of 6) to sample without any decent OHCs, these results indicated that hair cells are likely to undergo a massive alteration in the *Ftl*^−*/*−^ *HT* mice and that milder but significant alterations were also discernible in *Ftl*^−*/*−^ *LT* mice.

We then wondered whether the damages within the cochlea took place before or after the onset of hearing, which corresponds to the two-weeks period after birth in mice. Measure of ABR, right after the onset of hearing (P15-P16), demonstrated that the *Ftl*^−*/*−^ HT mice could be distinguished at this early stage (wave1_amp_ @ 16 kHz, 80 dB SPL, *Ftl*^−*/*−^ *HT*: 1 ± 0.6 µV versus *Ftl*^*+/+*^: 4.4 ± 1.5 µV and *Ftl*^−*/*−^ *LT*: 4.3 ± 1.6 µV; *P* = 7.10^−4^, one-way ANOVA test; post hoc Tukey’s test: *P* = 0.0014 in comparison to *Ftl*^*+/+*^ and *Ftl*^−*/*−^ *LT* mice, and *P* = 0.99 for *Ftl*^*+/+*^ versus *Ftl*^−*/*−^ *LT* mice; Supplementary Fig. 1a–c). However, to minimize prolonged anesthesia, which led to increased mortality at P15, we preferred to take advantage of DPOAEs, to distinguish the phenotypes among the *Ftl*^−*/*−^ mice. The distribution of the DPOAEs amplitude could be fit with a single Gaussian function in the P15 wild-type mice (*P* = 0.06, Shapiro-Wilk test) against a double Gaussian function fit in the P15 homozygous mice, reflecting presumably the *Ftl*^−*/*−^ *LT* mice and *Ftl*^−*/*−^ *HT* mice (*P* = 1.10^−4^, Shapiro-Wilk test; Supplementary Fig. 1d–f). While the wild-type and the *Ftl*^−*/*−^ *LT* mice had similar DPOAEs, the *Ftl*^−*/*−^ *HT* mice had reduced DPOAEs (mean 2f_1 _− f_2_ @ f_2_ from 5 to 20 kHz: 15.1 ± 3.4 dB, 15.9 ± 3.7 dB and 2.7 ± 1.8 dB in 1-month-old *Ftl*^*+/+*^ mice, *Ftl*^−*/*−^ *LT* mice and *Ftl*^−*/*−^ *HT* mice, respectively; Kruskal-Wallis test *P* < 0.0001; post hoc Dunn’s test: *P* < 0.0001 for *Ftl*^*+/+*^ versus *Ftl*^−*/*−^ *HT, P* < 0.0001 for *Ftl*^−*/*−^ *LT* versus *Ftl*^−*/*−^ *HT* and *P* > 0.99 for *Ftl*^*+/+*^ versus *Ftl*^−*/*−^ *LT*; Supplementary Fig. 1g–i). Anatomical examination showed that the fraction of altered or missing hair cells in the *Ftl*^−*/*−^ *LT* mice was small at P15 (1.1% of missing IHCs; *P* = 1.10^−3^ in comparison to the *Ftl*^*+/+*^, 2-sample test for equality of proportions with continuity correction; 1.1% of altered OHCs, *P* = 6.10^−3^ in comparison to the *Ftl*^*+/+*^ and 0.5% of missing OHCs, *P* = 1.10^−5^ in comparison to the *Ftl*^*+/+*^; Supplementary Fig. 1j–t). Although one cochlea out of 5 showed hair cells with splayed and missing stereocilia (approximatively 20% and 15% of IHCs and OHCs with splayed stereocilia, respectively, and 10% of OHCs with missing stereocilia in this sample, Supplementary Fig. 1s, t), the overall proportion of altered or missing hair cells was also limited in the *Ftl*^−*/*−^ *HT* mice (3.3% of altered IHCs, *P* = 5.10^−3^ in comparison to the *Ftl*^*+/+*^; 1.4% of altered OHCs, *P* = 2.10^−5^ in comparison to the *Ftl*^*+/+*^ and 0.7% of missing OHCs, *P* = 7.10^−8^ in comparison to the *Ftl*^*+/+*^). These data suggest that the functional impairment precedes a substantial morphological defect and that the degeneration may not occur during development but rather starts at the onset of hearing.

### Modulation of the calcium current by Ftl

**Fig. 6:**
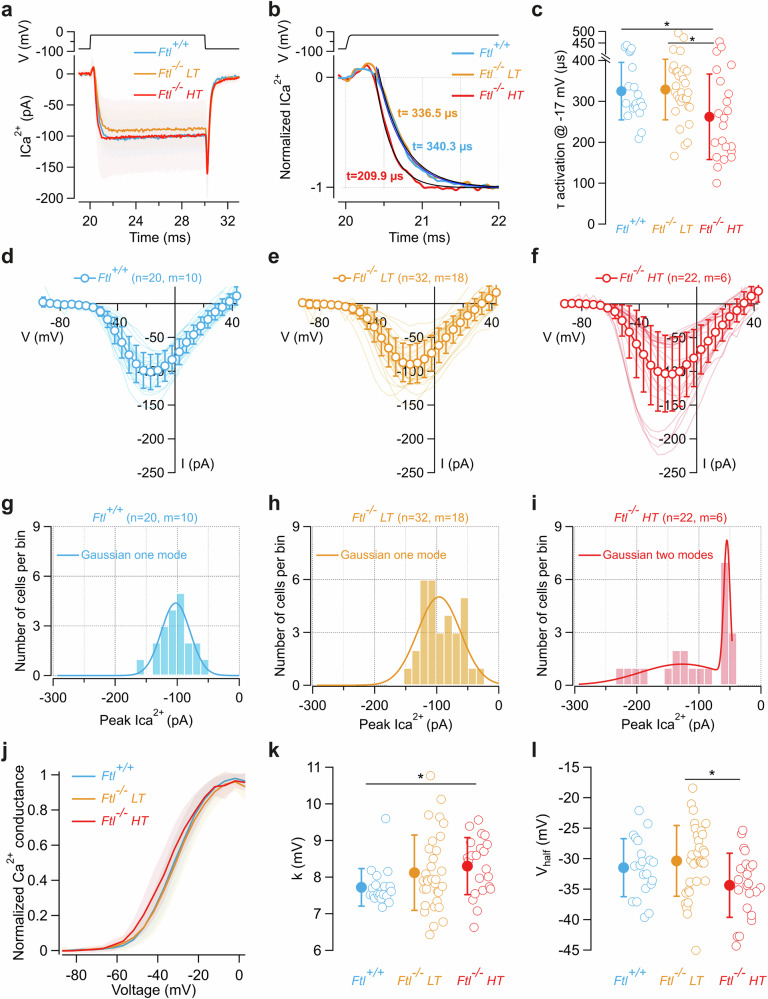
Alteration of the Ca^2+^-current in *Ftl*^−*/*−^*HT* mice. **a** Mean ± SD Ca^2+^ current (ICa^2+^) evoked by a 10 ms step depolarization to −17 mV from a holding potential of −87 mV in IHCs from *Ftl*^*+/+*^ mice (blue), *Ftl*^−*/*−^ *LT* mice (orange) and *Ftl*^−*/*−^ *HT* mice (red) between 15 and 21 postnatal days. **b** Normalized of mean ICa^2+^ shown in (**a**). *For Ftl*^*+/+*^ mice, *Ftl*^−*/*−^ *LT* mice and *Ftl*^−*/*−^ *HT* mice, respectively, Rs: 8.3 ± 0.3 MΩ, 7.9 ± 0.2 MΩ and 8.1 ± 0.3 MΩ; resting membrane capacitance: 11.1 ± 0.2 pF, 12.2 ± 0.2 pF and 10.8 ± 0.3 pF; resting current at holding potential (−87 mV): −16.6 ± 4 pA and −19.9 ± 2.7 pA and −21.7 ± 2.4 pA. Mean currents were fitted using a single exponential function over the 3 ms after the residual capacitive transients. **c** Mean ± SD activation kinetics time constant derived from the single exponential as described in (**b**). **d**–**f** Ca^2+^ current I/V relationships from *Ftl*^*+/+*^ mice (**d**), *Ftl*^−*/*−^ *LT* mice (**e**) and *Ftl*^−*/*−^ *HT* mice (**f**) between 15 to 21 postnatal days. Steady-state amplitude was measured as the average over the last 5 ms of the 10 ms test pulse. Thin lines represent individual recordings. Thick lines represent the mean ± SD. n and m indicate the number of recorded IHCs and mice, respectively. **g–i** Peak Ca^2+^ current distributions in *Ftl*^*+/+*^ mice (**g**), *Ftl*^−*/*−^ *LT* mice (**h**) and *Ftl*^−*/*−^ *HT* mice (**i**). Bin width: 13 pA. Histograms were fitted with a single Gaussian (thick lines) for *Ftl*^*+/+*^ mice (**g**) and *Ftl*^−*/*−^ *LT* mice (**h**). A two-Gaussian fit was used for the Ca^2+^ current distribution in *Ftl*^−*/*−^ *HT* mice (**i**). n and m indicate the number of recorded IHCs and mice, respectively. **j** Voltage dependence of calcium channel activation (activation curve) derived from the I/V relationships shown in (**a**). (**k**, **l**) Voltage of half-maximal calcium channel activation (V_half_, **k**) and voltage sensitivity of activation (slope, **l**) derived from fitting a Boltzmann function to the activation curves. Bar plots correspond to mean ± SD. Circles represent individual IHCs.

To know whether the morphological damages were limited to the stereocilia bundle or extended toward the basolateral side of the hair cells, we probed the IHC calcium influx through the voltage-gated calcium channels, which trigger exocytosis of glutamate filled synaptic vesicles and have been shown to be localized beneath the synaptic ribbon^19^. IHC calcium currents were elicited by step depolarization in the *Ftl*^*+/+*^ mice as well as in the *Ftl*^−*/*−^ mice (Fig. 6a). In IHCs from both *Ftl*^*+/+*^ mice and *Ftl*^−*/*−^ mice, the inward calcium current kinetics could be approximated using a single exponential fit (Fig. 6b). We observed differences in the calcium current kinetics from *Ftl*^−*/*−^ *HT* mice (Fig. 6b*, P* = 0.0116, one-way ANOVA test). The activation time constant was smaller in IHCs from *Ftl*^−*/*−^ *HT* mice (262.06 ± 104.4 µs @ − 17 mV; Fig. 6c) compared to the IHCs of the *Ftl*^*+/+*^ mice (324.9 ± 70.3 µs @ − 17 mV, *P* = 0.0445, post hoc Dunn’s test; Fig. 6c) and *Ftl*^−*/*−^ *LT* mice (328.8 ± 74.1 µs @ − 17 mV; *P* = 0.0139, post hoc Dunn’s test; Fig. 6c). The average current-voltage relationships were similar between the *Ftl*^*+/+*^ mice, *Ftl*^−*/*−^ *LT* mice and *Ftl*^−*/*−^ *HT* mice (*I*Ca^2+^ @ − 17 mV *Ftl*^*+/+*^: − 101.3 ± 26 pA, *n* = 20, *Ftl*^−*/*−^ *LT*: − 89.6 ± 28.7 pA, *n* = 32 and *Ftl*^−*/*−^ *HT*: − 103.9 ± 54.5 pA, *n* = 22; *P* = 0.4585, Kruskal-Wallis test; Fig. 6d–f). However, the IHC calcium currents amplitude seemed to be more variable in the *Ftl*^−*/*−^ *HT* mice. Indeed, the distribution of the peak calcium current of the *Ftl*^−*/*−^ *HT* mice did not follow a normal distribution (*P* = 0.0124, ShapiroWilk test; but could be approximated using a double Gaussian fit function (Fig. 6i). In contrast, the data from both *Ftl*^*+/+*^ mice (Fig. 6g) and *Ftl*^−*/*−^ *LT* mice (Fig. 6h) were normally distributed (*P* = 0.9645 and *P* = 0.2855, respectively; ShapiroWilk test; Fig. 6h) and could be fitted with the single Gaussian function. While the maximum peak of the calcium current was around − 100 pA in the *Ftl*^*+/+*^ mice and *Ftl*^−*/*−^ *LT* mice, two peaks at − 60 mV and − 130 mV were observed in *Ftl*^−*/*−^ *HT* mice (Fig. 6g–i).

Next, we analyzed the voltage-dependence of calcium channel activation by fitting a Boltzmann function to the fractional activation curves of the whole cell calcium current as described previously^20,21^. We found a significant (*P* = 0.0307, one-way ANOVA test) hyperpolarizing shift of 4 mV in the voltage of half-maximal calcium channel activation of *Ftl*^−*/*−^ *HT* mice (V_half_: − 34.37 ± 5.3 mV) compared *to Ftl*^−*/*−^ *LT* mice (V_half_: − 30.37 ± 5.8 mV; *P* = 0.0245, post hoc Dunn’s test; Fig. 6j, k). This shift was less prominent when comparing *Ftl*^−*/*−^ *HT* versus *Ftl*^*+/+*^ mice (V_half_: − 31.48 ± 4.8 mV; *P* = 0.1987, post hoc Dunn’s test; Fig. 6j, k). Moreover, we found a significant change in the voltage sensitivity of activation (slope; *P* = 0.0280, one-way ANOVA test). *Ftl*^−*/*−^ *HT* mice had a decreased voltage sensitivity (apparent from the bigger slope: 8.3 ± 0.78 mV) compared to *Ftl*^*+/+*^ mice (slope: 7.72 ± 0.51 mV; *P* = 0.0239, post hoc Dunn’s test; Fig. 6l). The sensitivity was comparable between *Ftl*^−*/*−^ *HT* and *Ftl*^−*/*−^ *LT* mice (slope: 8.12 ± 1.03 mV; *P* = 0.7725, post hoc Dunn’s test; Fig. 6l). Altogether, these data indicated that the calcium channel and/or auxiliary subunits may undergo structural changes in the *Ftl*^−*/*−^ *HT* mice leading to different current amplitude and kinetics.

### Neither biological sex, nor age nor a recessive allele contribute to hearing loss in Ftl KO mice

Hearing loss in a fraction of *Ftl*^−*/*−^ mice was puzzling. This led us to probe first any sex- dependence to the deleterious phenotype. Male and female mice were equally found within the *Ftl*^−*/*−^ *LT* mice (22 males and 21 females) and *Ftl*^−*/*−^ *HT* mice populations (7 males and 6 females). Splitting the audiograms, ABR and DPOAEs as a function of the sex showed that there were no differences between male and female mice within each mice population (Supplementary Fig. 2). For *Ftl*^−*/*−^ *LT* mice, male and female showed similar mean auditory thresholds (24.9 ± 4.1 dB SPL vs. 24.5 ± 3.8 dB SPL in male and female, respectively, *P* = 0.7275, unpaired t-test; Supplementary Fig. 2c), ABR wave1 amplitude at 16 kHz, 80 dB SPL (3.4 ± 1.6 µV vs 3.8 ± 1.3 µV in male and female, respectively; *P* = 0.4294, unpaired t-test; Supplementary Fig. 2i) and mean 2f_1_ − f_2_ amplitude from 5 to 20 kHz (20.2 ± 4.7 dB vs 22.4 ± 5.3 dB in male and female *Ftl*^−*/*−^ *LT* mice, respectively, *P* = 0.1602, unpaired t-test; Supplementary Fig. 2o). Similarly, male and female *Ftl*^−*/*−^ *HT* mice showed no differences in mean auditory thresholds (79.6 ± 15.4 dB SPL vs. 89.3 ± 18.1 dB SPL in male and female, respectively; *P* = 0.2297, two-tailed Mann-Whitney U test; Supplementary Fig. 2f), ABR wave1 amplitude at 16 kHz, 80 dB SPL (0.56 ± 0.5 µV vs 0.33 ± 0.3 µV in male and female *Ftl*^−*/*−^ *HT* mice, respectively, *P* = 0.1912, two-tailed Mann-Whitney U test; Supplementary Fig. 2l) and mean 2f_1_ − f_2_ amplitude from 5 to 20 kHz (2.8 ± 3.7 dB vs 0.3 ± 1.7 dB in male and female, respectively; *P* = 0.0883, two-tailed Mann-Whitney U test; Supplementary Fig. 2r). Thus, biological sex cannot account for the discrepancy among the *Ftl*^−*/*−^ mice.

Next, we postulated the hypothesis that the hearing loss in ferritin deficient mice develops over a variable time-course among individuals. Therefore, we carried out longitudinal assessment of the auditory function in 1 month- and 5 month-old wild-type and *Ftl*^−*/*−^ *LT* mice. Our results showed that the audiograms, ABR and DPOAEs did not change over 5 months between wild-type mice and *Ftl*^−*/*−^ *LT* mice (mean threshold shift: 2.4 ± 9.0 dB vs 2.2 ± 8.4 dB in *Ftl*^*+/+*^ and *Ftl*^−*/*−^ *LT* mice, respectively, *P* = 0.98, two-tailed *t*-test; ABR wave1 amplitude reduction @ 16 kHz, 80 dB SPL: 0.96 ± 0.8 µV vs 1.03 ± 1.5 µV in *Ftl*^*+/+*^ and *Ftl*^−*/*−^ *LT* mice, respectively, *P* = 0.674, two-tailed Mann-Whitney U test; mean 2f_1 _− f_2_ reduction @ f_2_ from 5 to 20 kHz: 2.3 ± 5.3 dB vs 0.5 ± 8.7 dB in *Ftl*^*+/+*^ and *Ftl*^−*/*−^ *LT* mice, respectively, *P* = 0.517, two-tailed *t*-test; Supplementary Fig. 3). Thus, these data argued against a variable onset of hearing loss in the *Ftl*^−*/*−^ mice.

Additionally, one might claim that in the Ftl knockout mice colony, an unknown recessive allele of hearing loss that is not related to ferritin, is at heterozygous state. In this scenario, *Ftl*^−*/*−^ *HT* mice would carry two copies of the mysterious allele causing deafness, while *Ftl*^−*/*−^ *LT* mice would harbor only one copy and hence, show no noticeable auditory phenotype. Such hypothesis could explain the mendelian-like distribution we observed (77% of *Ftl*^−*/*−^ LT mice vs 23% *Ftl*^−*/*−^ HT mice, Fig. 1). To test this scenario, we crossed male *Ftl*^−*/*−^ *HT* mice with female *Ftl*^−*/*−^ *HT* mice. If the hypothesis holds true, the litter should be exclusively populated by *Ftl*^−*/*−^ *HT* mice. However, the litter obtained with the aforementioned crossing was populated with 8 *Ftl*^−*/*−^ *LT* mice and only one *Ftl*^−*/*−^ *HT* mice (Supplementary Fig. 4), excluding therefore any potential recessive allele that would be responsible of the low penetrance of the phenotype.

### The *Ftl*^−*/*−^*LT* mice are vulnerable to noise exposure

**Fig. 7:**
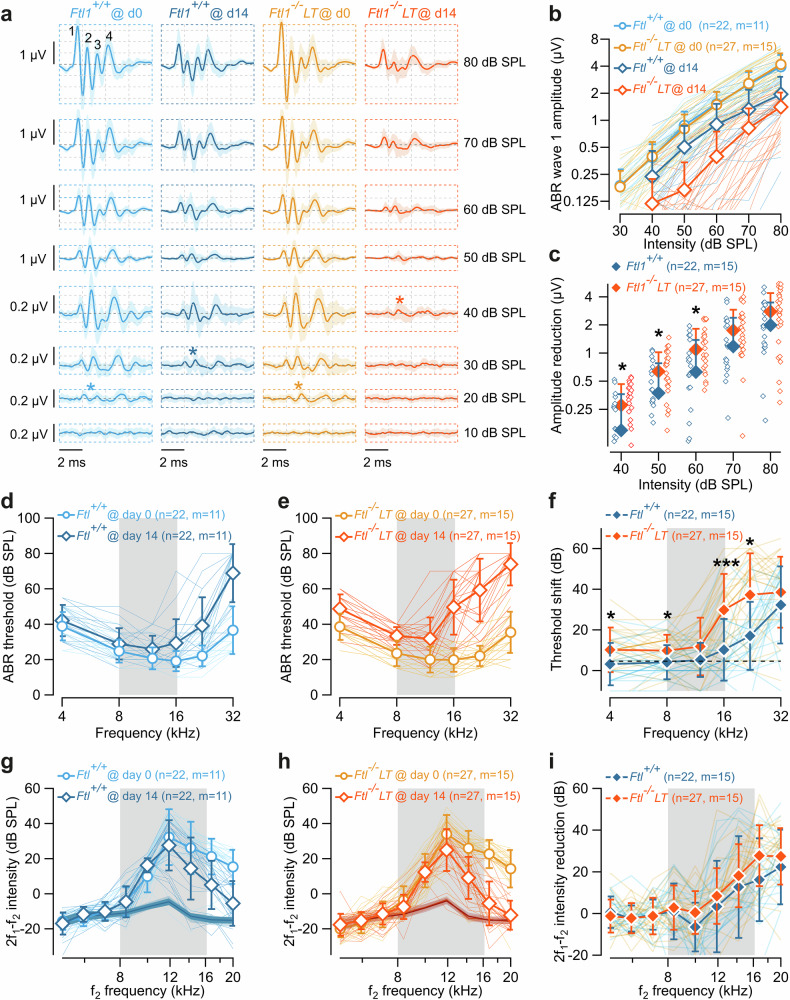
Vulnerability to noise exposure of *Ftl*^−*/*−^*LT* mice. **a** Mean ABR in 2-month-old wild-type mice (*Ftl*^*+/+*^, blue) and ferritin light chain knock-out low-threshold mice (*Ftl*^−*/*−^ *LT*, orange) before (d0) and two-weeks after (d14) an octave-band (8–16 kHz) noise exposure at 85 dB SPL during 8 h. Stars indicate the threshold-level. **b** Input-output function and (**c**) wave 1 ABR amplitude reduction derived from ABR in A. **d**, **e** Audiograms, (**f**) threshold shift, **g**, **h** DPOAEs and (**i**) DPOAEs amplitude reduction between the two genotypes before and two-weeks the octave-band noise exposure. Thin lines and symbols correspond to individual recordings. Thick lines and thick symbols indicate the mean ± SD. n and m indicate the number of recorded cochlea and mice, respectively. For *Ftl*^*+/+*^ mice, m = 4 males and 7 females; for *Ftl*^−*/*−^ *LT* mice, m = 7 males and 8 females.

Given that ferritin regulate the intracellular iron to prevent any reactive oxygen species production through the Fenton reaction, we wondered whether additional noxious factor, known to produce free radicals such as noise trauma^22^, would degrade the auditory function in the *Ftl*^−*/*−^ *LT* mice. Under this hypothesis, the presence of low hearing threshold and high hearing threshold Ftl^−/−^ mice in the same litter could result from differences in their exposure to noise. To test this hypothesis, we exposed wild-type mice and *Ftl*^−*/*−^ *LT* mice of either sex to 85 dB SLP, 8 kHz centered noise band during 8 h. Two weeks after the noise exposure, we observed a significantly larger threshold shift in the *Ftl*^−*/*−^ *LT* mice in comparison to the wild-type mice (mean threshold shift: 12.0 ± 7.4 dB vs. 22.9 ± 11.7 dB, respectively, *P* = 0.0005, two-tailed Mann Whitney U test; Fig. 7d, e, f). In both genotypes, the threshold shifts were predominantly localized toward the high-frequency of stimulation, consistent with the 8 kHz centered noise band exposure (threshold shift: 4.1 ± 8.4 dB @ 8 kHz and 10.2 ± 15.1 dB @ 16 kHz in the *Ftl*^*+/+*^ mice; 9.8 ± 7.7 dB @ 8 kHz and 29.8 ± 17.6 dB @ 16 kHz in the *Ftl*^−*/*−^ *LT* mice, Fig. 7f). While the amplitude of ABR wave 1 at 80 dB SPL was significantly reduced for both *Ftl*^*+/+*^ and *Ftl*^−*/*−^ *LT* mice after noise trauma, the magnitude of the reduction was similar for both groups. Yet, *Ftl*^−*/*−^ *LT* mice had a larger reduction of the first ABR wave 1 amplitude for low-intensity of stimulation (reduction in the ABR wave 1 amplitude @ 16 kHz, 50 dB SPL: 0.37 ± 0.4 µV vs. 0.63 ± 0.38 µV in *Ftl*^*+/+*^ and *Ftl*^−*/*−^ *LT* mice, respectively, *P* = 0.0426, two-tailed Mann-Whitney U test; Fig. 7a–c). Finally, while both genotypes showed reduced DPOAEs in the frequency regions above 12 kHz, the reduction was significantly larger for *Ftl*^−*/*−^ *LT* mice in DPOAEs @ f2 = 16.8 kHz (reduction in DPOAEs @ f2 = 16.8 kHz: 16.2 ± 19.8 dB in the *Ftl*^*+/+*^ mice; 27.7 ± 14.6 dB in the *Ftl*^−*/*−^ *LT* mice; *P* = *0.0237*, two-tailed *t*-test; Fig. 7g–i). Altogether, these results showed that the ferritin-deficient mice appeared to be more vulnerable to noise exposure than the wild-type. However, the noise-induced hearing loss in *Ftl*^−*/*−^ *LT* mice does not fully recapitulate the phenotype of *Ftl*^−*/*−^ *HT* mice, as the reductions in ABR and DPOAEs did not reach those observed in *Ftl*^−*/*−^ *HT* mice.

**Fig. 8:**
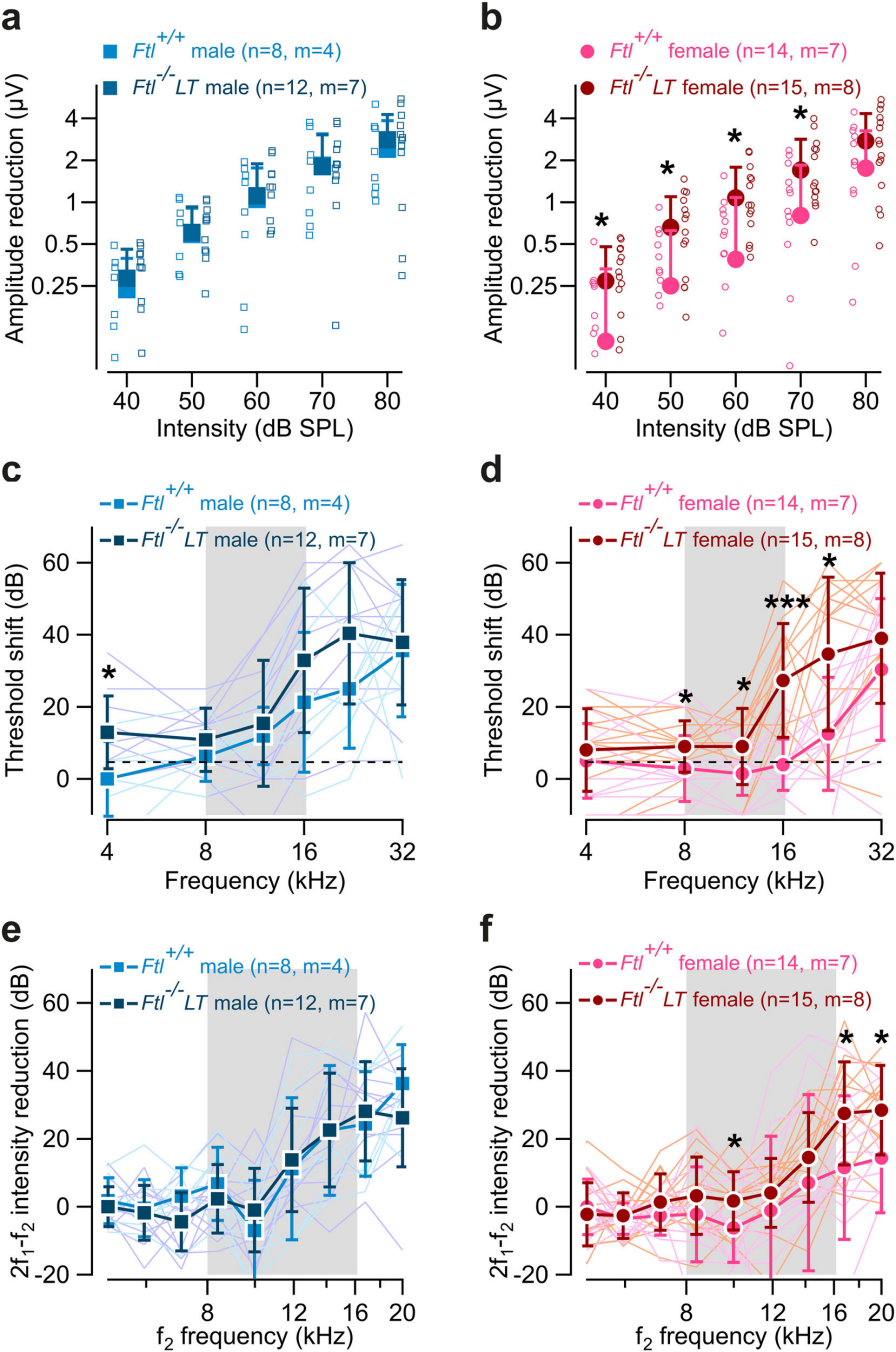
Vulnerability to noise exposure of *Ftl*^−^^*/*^^−^*LT* mice as a function of sex. **a**, **b** Wave 1 ABR amplitude reduction, (**c**, **d**) threshold shift and (**e**, **f**) DPOAEs amplitude reduction in 2-month-old male *Ftl*^*+/+*^ mice and *Ftl*^−*/*−^ *LT* mice (blue, **a**, **c**, **e**) and in 2-month-old female *Ftl*^*+/+*^ mice and *Ftl*^−*/*−^ *LT* mice (magenta, **b**, **d**, **f**) before and two-weeks after an octave-band (8–16 kHz) noise exposure at 85 dB SPL during 8 h. Thin lines and symbols correspond to individual recordings. Thick lines and thick symbols indicate the mean ± SD. n and m indicate the number of recorded cochlea and mice, respectively.

However, we wondered whether the noise exposure consequences could differ depending on the sex of *Ftl*^−*/*−^ *LT* mice given males are more susceptible to noise trauma than females^23–27^. We therefore split the audiograms, ABR and DPOAEs as a function of sex. Surprisingly, we found no difference in the auditory parameters after noise exposure between male *Ftl*^*+/+*^ mice and male *Ftl*^−*/*−^ *LT* mice. Both male wild-type and male *Ftl*^−*/*−^ *LT* mice were susceptible to noise exposure (mean threshold shift: 16.6 ± 6 dB vs 25.1 ± 13.6 dB in *Ftl*^*+/+*^ mice and *Ftl*^−*/*−^ *LT* mice, respectively, *P* = 0.1208, two-tailed *t*-test; reduction in the ABR wave 1 amplitude @ 16 kHz, 50 dB SPL: 0.58 ± 0.38 µV vs 0.6 ± 0.3 µV in *Ftl*^*+/+*^ mice and *Ftl*^−*/*−^ *LT* mice, respectively, *P* = 0.9066, two-tailed *t*-test; reduction in DPOAEs @ f2 = 16.8 kHz: 24.4 ± 15.4 dB in the *Ftl*^*+/+*^ mice; 28.1 ± 14.6 dB in the *Ftl*^−*/*−^ *LT* mice; *P* = 0.5922, two-tailed *t*-test; Fig. 8a, c, e). In contrast, female wild-type mice were more resistant to noise-exposure as evident by significantly larger threshold shift in the female *Ftl*^−*/*−^ *LT* mice (mean threshold shift: 9.35 ± 7 dB vs 21.1 ± 10.1 dB in *Ftl*^*+/+*^ mice and *Ftl*^−*/*−^ *LT* mice, respectively; *P* = 0.0011, two-tailed *t*-test; Fig. 8d). We also noticed a greater decrease of the first ABR wave 1 amplitude in the female *Ftl*^−*/*−^ *LT* mice in comparison to the female wild-type mice (reduction in the ABR wave 1 amplitude @ 16 kHz, 50 dB SPL: 0.25 ± 0.37 µV vs 0.66 ± 0.43 µV in *Ftl*^*+/+*^ mice and *Ftl*^−*/*−^ *LT* mice, respectively, *P* = 0.0119, two-tailed *t*- test; Fig. 8b). Finally, there was a significant difference in the DPOAEs loss in female *Ftl*^−*/*−^ *LT* mice (reduction in DPOAEs @ f2 = 16.8 kHz: 11.5 ± 21.1 dB in the *Ftl*^*+/+*^ mice; 27.4 ± 15.1 dB in the *Ftl*^−*/*−^ *LT* mice; *P* = *0.0267*, two-tailed *t*-test; Fig. 8f). Thus, female *Ftl*^−*/*−^ *LT* mice exposed to noise would show hearing loss with features comparable, to some extent, to *Ftl*^−*/*−^ *HT* mice.

## Discussion

In our study, we showed that the loss of the ferritin light-chain, known to chelate iron, results in hearing deficits, damage of hair cells and impaired calcium current in a fraction of *Ftl*^−*/*−^ mice. In addition, loss of Ftl leads to a higher susceptibility to acoustic trauma. Thus, our study suggest that iron homeostasis may be critical to the physiology of auditory sensory cells, particularly in noxious environments.

### Ftl is required for hair cell function and survival

By converting and storing iron under its ferric state, ferritin prevents the formation of hydroxyl radicals^6^. As a consequence, the elimination of the ferritin heavy- or light-chain triggers cellular oxidative stress, which eventually impairs cellular function [9]. In our study, the genetic deletion of Ftl in mice resulted to two distinct phenotypes: a group with an auditory deficit (*Ftl*^−*/*−^ *HT* mice) and a group with preserved auditory function (*Ftl*^−*/*−^ *LT* mice). This finding is consistent with the first observation of the *Ftl*^−*/*−^ phenotype, in which the authors observed tilting head and circling behavior among the *Ftl*^−*/*−^ mice, indicative of a potential alteration in the inner ear^16^. It is also consistent with the expression of Ftl in several cochlear cells type. Indeed, previous studies have reported the expression of Ftl in the IHCs and OHCs^28^, in the non-sensory cells such as the pillar and Deiters cells^28^ as well as in the stria vascularis^29,30^. In the *Ftl*^−*/*−^ *HT* mice, the elevation in threshold is associated with the reduction of the DPOAEs, consistent with the degeneration of the OHCs and the vulnerability of these sensory cells to oxidative stress^31^. In addition, the splayed stereocilia in some IHCs may further contribute to the auditory deficit. Indeed, the disorganization of the stereocilia in the IHCs is known to elevate the auditory threshold and reduce the ABR at high-intensity of sound-stimulation^32,33^. Of note, the IHCs were found to be more resistant than the OHCs, which is consistent with a previous study showing that H_2_O_2_-induced oxidative stress preferentially damaged the OHCs^34^.

In the *Ftl*^−*/*−^ *HT* mice, the anatomical defect in hair cells contrasts with the preserved morphology in the stria vascularis. This result is striking because the stria vascularis contains blood vessels and has been shown to accumulate ferritin-like particles and iron^35^. These observations have led the authors to postulate the hypothesis that ferritin chelates iron from dying erythrocytes to prevent excessive oxidative stress in the stria vascularis^35^. The lack of morphological damage within the stria vascularis might be explained by a differential expression of the light and heavy ferritin chain^36^. Under this hypothesis, Ftl would be more abundant in hair cells while Fth1 would predominate in the stria vascularis. However, we still observed a reduction in the endocochlear potential, indicating the alteration in the stria vascularis activity. The fall in EP should lead to a reduced transducer current in hair cell and diminishes the amplitude of the hair cell receptor potential to acoustic stimulation, contributing further to the hearing loss^37^. In addition, the small EP might be solely responsible of the degeneration of hair cells^38^. This hypothesis is, however, unlikely as mutant mice with reduced EP still have decent hair cells^39^ and it has recently been show that EP above 18 mV suffices to maintain hair cell survival^38^. Given the lack of morphological alteration in the stria vascularis, loss of Ftl may lead to the rise of hydroxyl radicals within the stria vascularis, impeding in turn the activity of ion channels such as Kir4.1 or ClC-K, required to generate large EP^40,41^. Alternatively, the collapse of the OHCs may yield to a reduced EP due to an insufficient recycling of potassium toward the stria vascularis^42,43^. Taken together, both defective IHCs and OHCs with the EP reduction contribute to the hearing loss in the *Ftl*^−*/*−^ *HT* mice.

In addition, damages in the ferritin-deficient mice were not only restricted to the OHCs and IHCs stereocilia. We noted changes in calcium current kinetics, amplitudes and voltage-dependence from the *Ftl*^−*/*−^ *HT* mice. Therefore, additional defects might take place at the IHC synapses. Here again, the increase of hydroxyl radicals within the IHCs could account for changes in synaptic calcium dynamics. Indeed, hydrogen peroxide application, known to produce hydroxyl radical, have been reported to modify the calcium current amplitude in a concentration-dependent manner^44^ and to accelerate the channel opening transition^45^ presumably through the oxidation of cysteine residues, that would alter the calcium channel structure through disulfide bonds. In this framework, preventing the formation of hydroxyl radical near the calcium channel is of prime importance to maintain the synaptic activity intact.

### Mechanisms underlying the divergent phenotype among the Ftl colony

Although alterations in systemic iron homeostasis has been associated with auditory deficits in human^46,47^, mutations in the *FTL* gene have not, to date, been reported as a cause of hearing impairment^48,49^. The cellular machinery could easily cope the lack of Ftl to regulate the oxidative stress related to iron metabolism unless exposed to additional stress. For instance, the heterozygous *Fth*^*+/*−^ mice show retinal degeneration in response to excessive light^50^. Mutant mice for pejvakin, which is involved in the regulation of oxidative stress by peroxisome, exhibit variable degree of hearing loss depending of the litter size^51^. Given the vulnerability of the pejvakin mutant mice to sound exposure, the authors suggested that pup vocalizations in large litters generate intense noise that would harm auditory function^51,52^. We found that *Ftl*^−*/*−^ *LT* mice are more susceptible to noisy environment than the wild-type mice. This could be due to the increase in hydroxyl radicals that has been shown to occur after noise exposure^22^. Thus, in our study, individuals exposed to daily and repeated noise may acquire the *Ftl*^−*/*−^ *HT* phenotype. However, this hypothesis has to be taken with caution. First, the noise paradigm we used in our study provokes threshold shift only in female given the larger susceptibility of wild-type male to noise^23–25^. The sex-dimorphism regarding the noise vulnerability has been previously reported and involved OHC and ribbon synapses protection through estradiol signaling^26,27^. Because our acoustic exposure paradigm makes the noise consequence indistinguishable between wild-type and *Ftl*^−*/*−^ male mice, further experiments with different protocols are needed to corroborate the hypothesis that the noise exposure equally affects male and female *Ftl*^−*/*−^ *LT* mice^53^. Second, noise within the animal facility would affect all the individuals in the litter rather than in a proportion of mice. Finally, a fraction of *Ftl*^−*/*−^ *HT* mice is observed right after the onset of hearing, corresponding to a period in which the cochlea did not reach full maturity, and hence is still insensitive to noise trauma^54^. Thus, other factors may be responsible for the hearing loss in a proportion of *Ftl*^−*/*−^ mice. For instance, the influence of the genetic background on the phenotype cannot be fully discarded^55^. Indeed, the C57BL/6 J is known to carry the Cdh23-ahl allele^56^, which may impose additional stress on the auditory system. Further experiments, which consist to study the Ftl deletion in another strain or to carry-out whole genome sequencing, are required to test this hypothesis. In addition, loss of Ftl leads to embryonic lethality in approximately 50% of the expected *Ftl*^−*/*−^ embryos^16^. The authors raised the hypothesis that the light-chain plays a pivotal role in mouse embryogenesis^57^ and that ferritin heavy chain may rescue, albeit not completely, the loss of the light chain in mouse^16^. A similar scheme may apply to the cochlea. While further experiments are required to solve the low penetrance of the phenotype in the ferritin-deficient mice, our study suggests that the coordinated regulation of iron homeostasis is a prerequisite for the cochlea to operate at best conditions.

## Materials and methods

### Ethical approval

We have complied with all relevant ethical regulations for animal use. Experiments were carried out in accordance with the animal welfare guidelines 2010/63/EC of the European Communities Council Directive regarding the care and use of animals for experimental procedures. Animals were housed in facilities accredited by the French “Ministère de l’Agriculture et de la Forêt” (Agreement C-34-172-36) and the experimental protocol was approved (Authorization CEEA-LR- 12111) by the Animal Ethics Committee of Languedoc-Roussillon (France).

### Animals

We studied ferritin light chain knockout mice (*Ftl*^−*/*−^*)* of either sex, which have been previously described (Li et al., 2015). In brief, ferritin knock-out mice were generated by the removal of exons 1 and 2, leading to the complete loss of the light chain subunit^16^. *Ftl*^−*/*−^ mice founders (MGI:6115891) were provided by the laboratory of Ruben Vidal (Indianapolis, USA). The *Ftl*^−*/*−^ knockout colony was annually refreshed by crossing *Ftl1*^−*/*−^ mice with C57BL/6 J mice (C57BL/6JRj from Janvier Labs). Experimental cohorts were generated through *Ftl*^*+/*−^ x *Ftl*^*+/*−^ crossing and *Ftl*^−*/*−^ x *Ftl*^−*/*−^ crossing. Sex of the animals was only recorded to assess sex-dependence of the phenotype between *Ftl*^−*/*−^ *LT* and *Ftl*^−*/*−^ *HT* mice.

### Genotyping

Genotypes of mice was determine by Polymerase Chain Reaction (PCR) analysis of genomic DNA using FastStart PCR Master Mix (Roche Applied Science, Penzberg, Germany) as previously described^16^. In brief, DNA from tail or toe biopsies was extracted using KAPA Express Extract Kit (KK7103, Roche Applied Science, Penzberg, Germany). The PCR was conducted with the following thermal cycle program: 1 cycle of 95 °C for 10 min; 39 cycles of 94 °C for 40 s, 58 °C for 30 s, 72 °C for 40 s; final elongation step at a single cycle 72 °C for 7 min. The PCR primers were the following: the forward primer: 5’- CCTCAGCTCCGGATTGGT-3’ and the reverse primer: 5’-GTTCCGTTCAAACACTGTTG-3’ were used to detect the wild-type allele (180 bp). The forward primer and the reverse primer: 5’-GCACAGGAAAAGTGGGCACAGT-3’ were used to detect the knock-out allele (500 bp). Amplicons were visualized on 2% agarose gels (ClearSight DNA Stain, Euromedex, Souffelweyersheim, France).

### In vivo recordings

Mice (P15 to 5-month-old) were anesthetized by an intraperitoneal injection of a mixture of tiletamine–zolazepam (Zoletil 50; 40 mg/kg) and xylazine (Rompun 2%; 3 mg/kg). The rectal temperature was measured with a thermistor probe and maintained at 37.1 °C ± 1 °C, using a heated underblanket (Homeothermic Blanket Systems, Harvard Apparatus). Heart rate was monitored via EKG electrodes. Within 20 min after the end of the physiological session, animals were sacrificed by cervical dislocation still under deep anaesthesia. For auditory brainstem response (ABR), the acoustic stimuli consisted of 10 ms tone bursts, with an 8-ms plateau and 1 ms rise/fall time, delivered at a rate of 20.4/s with alternate polarity by a JBL 2426H loudspeaker in a calibrated free field. Stimuli were presented by varying intensities from 100/80 dB SPL to 0 dB SPL, in 10/5 dB step. Stimuli were generated and data acquired using MATLAB (MathWorks, Natick, MA, USA) and LabVIEW (National Instruments, Austin, TX, USA) software. The potential difference between vertex and mastoid intradermal needles was amplified (20000 times, VIP-20 amplifier), sampled (at a rate of 50 kHz), filtered (bandwidth of 0.3–3 kHz) and averaged (650 times). Data were displayed using LabVIEW software and stored on a computer (Dell T7400). ABR thresholds were defined as the lowest sound intensity, which elicits a clearly distinguishable response. For distortion product otoacoustic emissions recordings (DPOAEs), an ER-10C S/N 2528 probe (Etymotic Research), consisting of two emitters and one microphone, was inserted in the left external auditory canal. Stimuli were two equilevel (65 dB SPL) primary tones of frequency f_1_ and f_2_ with a constant f_2_/f_1_ ratio of 1.2. The distortion 2f_1 _− f_2_ was extracted from the ear canal sound pressure and processed by HearID auditory diagnostic system (Mimosa Acoustic) on a computer (Hewlett Packard). The probe was self-calibrated for the two stimulating tones before each recording. f_1_ and f_2_ were presented simultaneously, sweeping f_2_ from 20 kHz to 2 kHz by quarter octave steps. For each frequency, the distortion product 2f_1 _− f_2_ and the neighbouring noise amplitude levels were measured and expressed as a function of f_2_. For endocochlear potential, after a ventrolateral approach of the cochlea, the bone over the basal turn of the scala media was gently shaved and a small fenestra was made through the thinned bone. A glass microelectrode (tip diameter 0.1–0.5 µm) was filled with 3 M KCl and connected to a direct current amplifier (WPI, model 773 A, Sarasota, FL, USA). The microelectrode was positioned under visual control at a point and angle appropriate to be passed through the fenestra and into the scala media to record the endocochlear potential. The Ag/AgCl reference wire was placed in the animal’s neck musculature.

### Noise exposure

Awake and unrestrained 2-month-old mice of either sex were placed in an exposure chamber (3 to 4 mice per session). A suspended horn loudspeaker delivered 8–16 kHz noise-band at 85 dB SPL during 8 h. Food and water were available to mice within the exposure chamber. ABR and DPOAEs were recorded one day before exposure and 14 days after noise exposure. 3 and 4 sessions were performed for *Ftl*^*+/+*^ mice and *Ftl*^−*/*−^ *LT*, respectively.

### Longitudinal assessment of the auditory function

ABR and DPOAEs were recorded in *Ftl*^*+/+*^ mice and *Ftl*^−*/*−^ *LT* mice at 1 month of age. Mice were then housed in an adjacent animal facility room, and their auditory function was re-evaluated at 5 months of age.

### Patch-clamp recordings

After cervical dislocation of mice (from P15 to 1 month of age), IHCs of the apical coil of freshly dissected organs of Corti were patch-clamped at their basolateral face at room temperature in tight whole-patch configurations. The dissection solution contained the following in mM: 5.36 KCl, 141.7 NaCl, 1 MgCl_2_-6H_2_O, 0.5 MgSO_4_-7H_2_O, 10 HEPES and 10 D-glucose. The extracellular solution contained the following in mM: 105 NaCl, 35 tetraethylammonium-Cl (TEA-Cl), 2.8 KCl, 1 MgCl2, 10 HEPES, 1 CsCl, 2 CaCl2, and 10 D-glucose. The pipette solution for tight whole-cell patch-clamp recordings contained the following in mM: 135 Cs-glutamic acid, 10 TEA-Cl, 10 4-aminopyridine, 1 MgCl_2_-6H_2_O, 2 Mg-ATP, 0.3 Na-GTP, 10 HEPES and 0.1 EGTA. Solutions were adjusted to pH 7.3 and had osmolarities between 290 and 310 mOsm/kg H_2_O. All chemicals were obtained from Sigma (St. Louis, MO, USA). Patch pipettes were pulled from borosilicate glass capillaries (Kwik Fil, WPI, Worcester, MA, USA) with a two-step vertical puller PIP 6 (HEKA Elektronik, Lambrecht, Germany) and coated with silicone elastomer (Sylgard). Calcium currents were low-pass filtered at 3 kHz and sampled at 50 kHz, and were isolated using P/n protocols (10 leak pulses with amplitudes of 20% of the original pulse from a holding potential of − 117 mV. Cells that displayed a membrane current exceeding − 60 pA at − 87 mV were discarded from the analysis. No Rs compensation was applied. All voltages were corrected for liquid junction potentials calculated between pipette and bath ( − 17 mV). An EPC-10 amplifier (HEKA Elektronik) controlled by Patchmaster software (HEKA Elektronik) was used for all measurements.

### Electron microscopy

Scanning (SEM) and transmission (TEM) electron microscopy were done for the anatomical examination of cochlear hair cells. For both techniques, the animals (P18 to 1-month-old) were decapitated under deep anaesthesia (pentobarbital, 50 mg/kg), their cochleae were removed from the temporal bone. For SEM, PBS washed cochlea were fixed with 2.5% glutaraldehyde in phosphate buffer (pH 7.2) for 2 h at room temperature, followed by washing in phosphate buffer. The bony capsule of the cochlea was dissected out, and the stria vascularis as well as the tectorial and Reissner’s membranes were removed. Fixed cochleae were dehydrated using a graded ethanol series (15–100%), followed by critical point drying with CO_2_. Subsequently, the samples were sputter coated with an approximatively 10 nm thick gold film and then examined under a scanning electron microscope (Hitachi S4000) using a lens detector with an acceleration voltage of 10 kV at calibrated magnifications. For TEM, the cochleae were perfused with a solution of 2.5% glutaraldehyde in PHEM buffer (1X, pH 7.4) and immersed in the same fixative for 1 h at room temperature and overnight at 4 °C. The samples were then rinsed in PHEM buffer and post-fixed in a 0.5% osmic acid for 2 h in the dark at room temperature. After two rinses in PHEM buffer, the samples were dehydrated in a graded series of ethanol solutions (30–100%). The samples were embedded in EmBed 812 using an Automated Microwave Tissue Processor for Electronic Microscopy, Leica EM AMW. Semithin (1 µm) and thin transverse sections (70 nm) were collected at different levels of each block (Leica-Reichert Ultracut E). Semithin sections were counterstained with toluidine blue and observed using Nanozoomer 1 Hamamatsu. Thin sections were counterstained with uranyl acetate 1.5% in 70% Ethanol and lead citrate and observed using a Tecnai F20 transmission electron microscope at 200KV. Both SEM and TEM were conducted at the CoMET facility (MRI, INM, Montpellier, France).

### Statistics and reproducibility

Data were analysed using Igor Pro (WaveMetrics, Lake Oswego, OR, USA), MATLAB and R (R core team) software. All data are expressed as means ± standard deviation (SD). Statistical analysis was performed using GraphPad Prism 9. Data was tested for normal distribution and equality of variances (Saphiro-Wilk test and F test). Statistical significance was assessed with unpaired two-tailed *t*-test, Welch’s test or Mann-Whitney U test when two data sets were compared, one-way ANOVA test or Kruskal-Wallis test when three datasets were compared, or two-sample test for equality of proportions. Statistical analyses were conducted on the number (n) of cochleae used for in vivo recordings, or on the number (n) of hair cells for anatomical examinations and patch-clamp recordings. All samples were harvested from a pool of m mice, corresponding to the biological replicates. Sample sizes (n) and biological replicates (m) for each experiment are indicated in the figure legends. For experiments lacking formal statistical analysis, reproducibility was assessed across at least three independent biological replicates.

### Reporting summary

Further information on research design is available in the Nature Portfolio Reporting Summary linked to this article.

## Supplementary information

Transparent Peer Review file

Supplementary information

Description of Additional Supplementary files

Supplementary data

Reporting Summary

## Data Availability

Numerical source data for graphs can be found in Supplementary Data and all other data are available from the corresponding author on reasonable request.
