# Supplementary material for: Ferritin light-chain contributes to hair cells function and survival
Source: Commun Biol. 2026 Apr 18;9:839. doi: 10.1038/s42003-026-10069-3 (PMC13276406; doi:10.1038/s42003-026-10069-3)
Supplement: Supplementary file 2 — Supplementary information [file 42003_2026_10069_MOESM2_ESM.pdf]

Supplementary information for

Ferritin light-chain contributes to hair cells function and survival

Chloé P. Petit, Sahia Mahaman Bachir Dodo, Lina Maria Jaime-Tobon, Anne-Gabrielle Harrus, Cécilia Souyris, Antoine Picot, Jing Wang, Rémy Pujol, Frédéric Venail, Ruben Vidal, Jérôme Bourien, Jean-Luc Puel and Régis Nouvian

This PDF file includes:

Supplementary Figure 1:
Structure-function examination in P15 *Ftl*$^{-/-}$ mice

Supplementary Figure 2:
Auditory phenotype in *Ftl*$^{-/-}$ mice does not show any preferential sex dependence.

Supplementary Figure 3:
Ftl$^{-/-}$ *LT* mice do not show hearing loss over 5 months of age.

Supplementary Figure 4:
An additional recessive allele is unlikely to contribute to hearing loss in *Ftl* deficient mice.

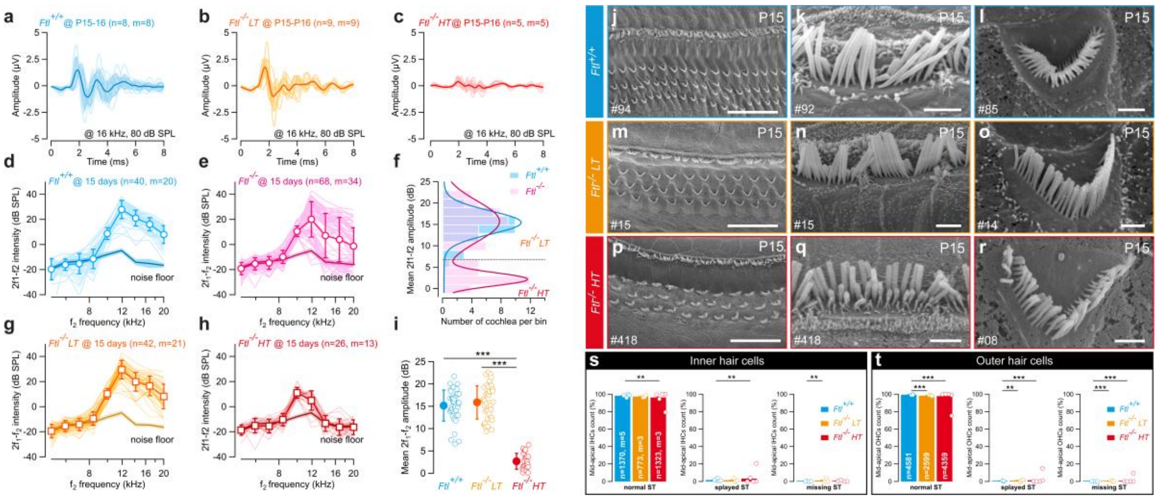

Supplementary Figure 1: Structure-function examination in P15 *Ftl*$^{-/-}$ mice. (**a-c**) ABR evoked by a 16 kHz tone burst at 80 dB SPL in P15-16 wild-type mice (*Ftl*$^{+/+}$, blue, **a**), ferritin light chain knock-out mice (*Ftl*$^{-/-}$ *LT*, orange, **b**; *Ftl*$^{-/-}$ *HT*, red, **c**). (**d, e**) DPOAEs in P15-old wild-type (*Ftl*$^{+/+}$, blue, **a**) and ferritin light chain knock-out mice (*Ftl*$^{-/-}$, magenta, **e**). Distribution of audiograms (**f**, bin width: 2 dB) shown in (**d**, **e**). Histograms were fitted with a single or two Gaussian (thick lines) for wild-type mice (*Ftl*$^{+/+}$, blue) and ferritin light chain knock-out mice (*Ftl*$^{-/-}$, magenta), respectively. The dashed black line segregates the *Ftl*$^{-/-}$*HT* mice from the *Ftl*$^{-/-}$ *LT* mice. (**g** and **h**) DPOAEs in ferritin light chain knock-out mice according to the distribution in (**f**). For (**a-e**, **g-h**) Thin lines correspond to individual recordings. Thick lines indicate the mean ± SD. n and m indicate the number of recorded cochlea and mice, respectively. (**i**) Mean of DPOAEs from wild-type mice (*Ftl*$^{+/+}$, blue) and ferritin light chain knock-out mice (*Ftl*$^{-/-}$ *LT*, orange and *Ftl*$^{-/-}$ *HT*, red). Circles correspond to individual measurements. (**j-r**) Scanning electron microscopy of the organ of Corti (**j**, **m** and **p**), stereocilia bundle from inner (**k**, **n** and **q**) and outer (**l**, **o** and **r**) hair cells at mid-apical turns from P15-old *Ftl*$^{+/+}$ mice, *Ftl*$^{-/-}$ *LT* mice and *Ftl*$^{-/-}$ *HT* mice. # indicates the animal's number. Scale bars: 20 µm in **j**, **m**, **p**; 2 µm in **k**, **l**, **q** and 1 µm: **l**, **o**, **r**. (**s**, **t**) Fraction of inner (**s**) and outer (**t**) hair cells harboring normal, splayed or missing stereocilia (ST). Percentages are shown with their SD. IHCs: inner hair cells; OHCs: outer hair cells. n and m indicate the number of hair cells examined and mice, respectively. Symbols represent individual cochleas.

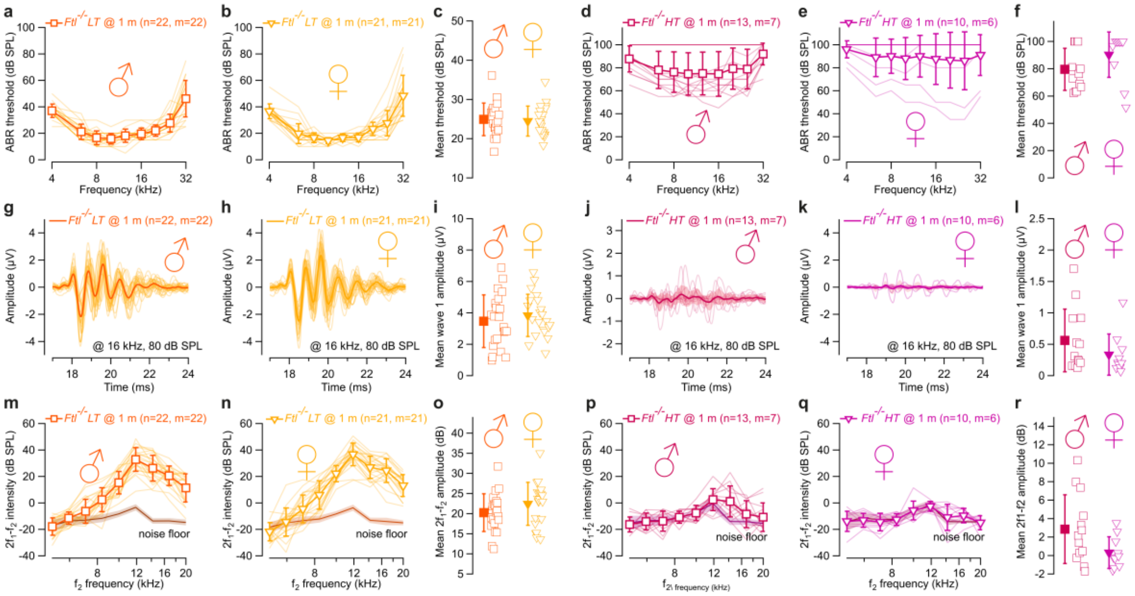

Supplementary Figure 2: Auditory phenotype in *Ftl*$^{-/-}$ mice does not show any preferential sex dependence. (**a-b**, **d-e**) audiograms, (**g-h**, **j-k**) ABR and (**m-n**, **p-q**) distortion products of otoacoustic emissions in male *Ftl*$^{-/-}$ *LT* mice (**a**, **g** and **m**), female *Ftl*$^{-/-}$ *LT* mice (**b**, **h** and **n**), male *Ftl*$^{-/-}$ *HT* mice (**d**, **j** and **p)** and female *Ftl*$^{-/-}$ *HT* mice (**e**, **k** and **q**) at 1 month of age. Thick lines indicate the mean ± SD. (**c**, **f**) mean threshold, (**i**, **l**) ABR wave I amplitude and (**o**, **r**) mean DPOAEs in male and female *Ftl*$^{-/-}$ *LT* mice (**c**, **i** and **o**) and *Ftl*$^{-/-}$ *HT* mice (**f**, **l** and **r**). n and m indicate the number of cochleas and mice, respectively.

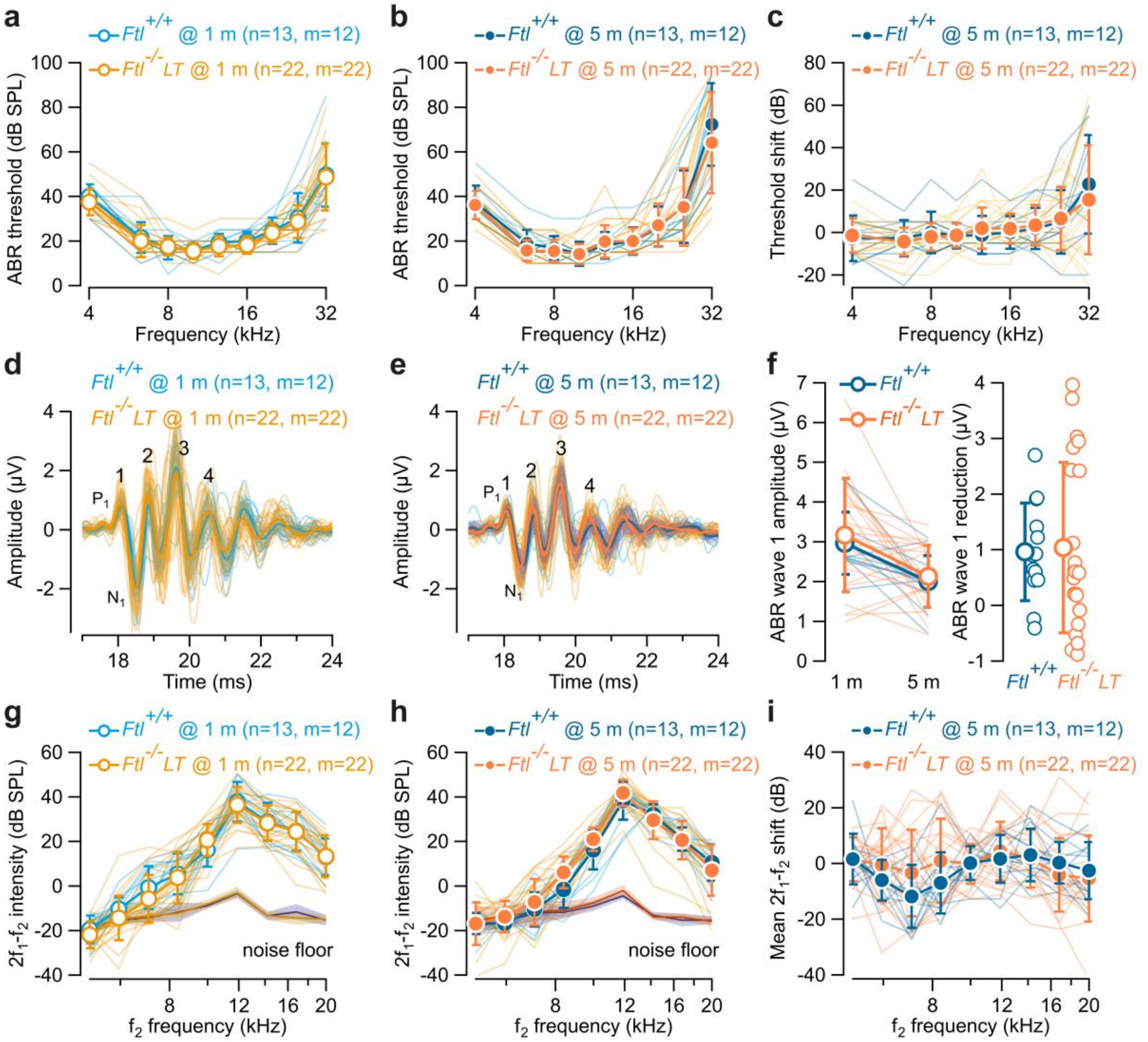

Supplementary Figure 3: *Ftl*$^{-/-}$ *LT* mice do not show hearing loss over 5 months of age. (**a**-**b**) audiograms, (**d**-**e**) ABR and (**g**-**h**) distortion products of otoacoustic emissions in wild-type *Ftl*$^{+/+}$ mice (blue) and knock-out mice *Ftl*$^{-/-}$ *LT* mice (orange) at 1 month (**a**, **d** and **g**) and 5 months (**b**, **e** and **h**) of age. Thick lines indicate the mean ± SD. (**c**, **f** and **i**) Threshold shift (**c**), ABR amplitude reduction (**f**) and DPOAEs reduction (**i**) over 5 months of age in wild-type *Ftl*$^{+/+}$ mice (blue) and knock-out mice *Ftl*$^{-/-}$ *LT* mice (orange). n and m indicate the number of cochleas and mice, respectively.

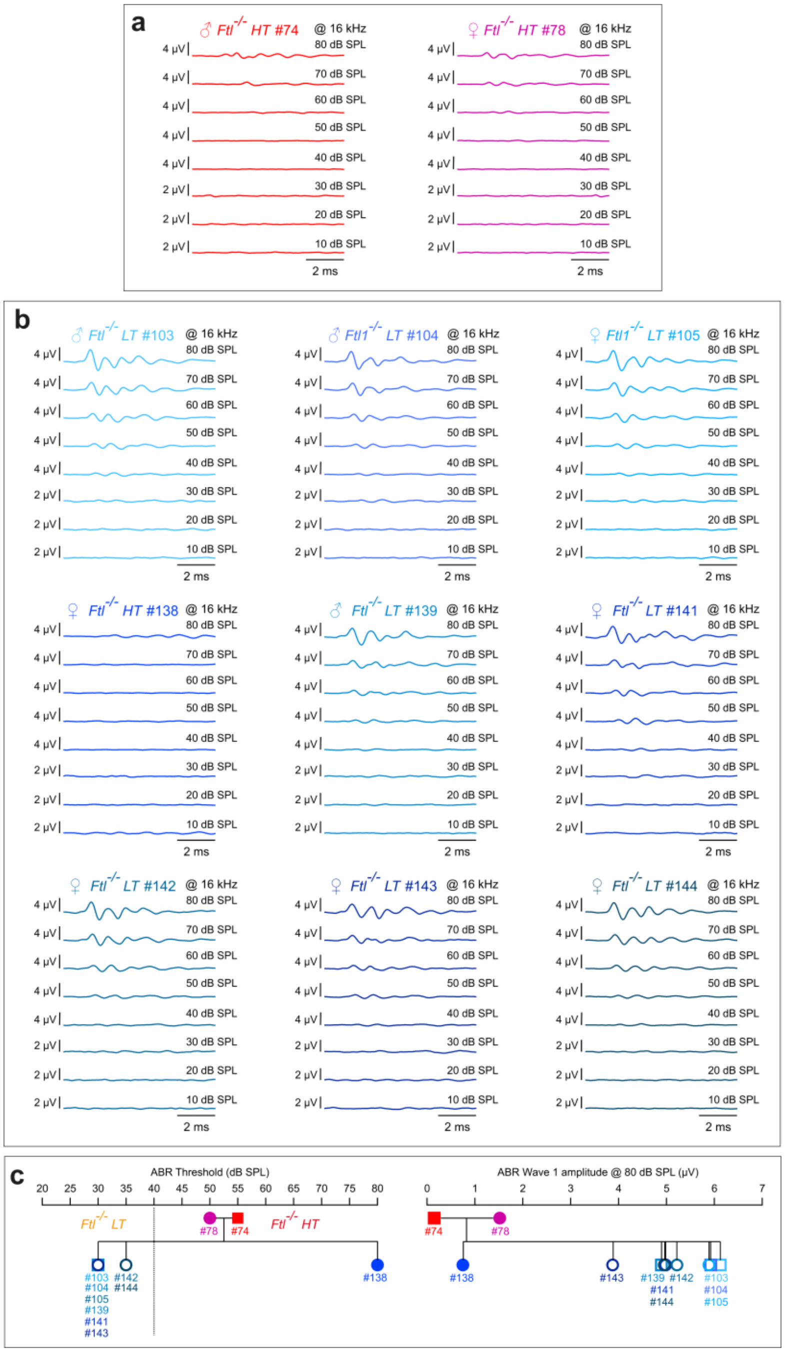

Supplementary Figure 4: An additional recessive allele is unlikely to contribute to hearing loss in *Ftl* deficient mice. (**a**) Mean ABR of one-month-old male (red) and female (purple) *Ftl*$^{-/-}$ *HT* mice breeders evoked by 16 kHz tone burst from 10 to 80 dB SPL. (**b**) Mean ABR of the offspring evoked by 16 kHz tone burst from 10 to 80 dB SPL. (**c**) ABR threshold and wave 1 amplitude of *Ftl*$^{-/-}$ *HT* breeders and their offspring probed at one month of age.
