# Supplementary material for: Ferritin light-chain contributes to hair cells function and survival
Source: Commun Biol. 2026 Apr 18;9:839. doi: 10.1038/s42003-026-10069-3 (PMC13276406; doi:10.1038/s42003-026-10069-3)
Supplement: Supplementary file 1 — Transparent Peer Review file [file 42003_2026_10069_MOESM1_ESM.pdf]

nature portfolio

Peer Review File

Ferritin light-chain contributes to hair cells function and survival

Corresponding Author: Dr Régis Nouvian

This file contains all reviewer reports in order by version, followed by all author rebuttals in order by version.

Version 0:

Reviewer comments:

Reviewer #1

(Remarks to the Author)
General:

The MS entitled

‚Ferritin light-chain contributes to hair cells function and survival‘

by Chloé P. Petit, Sahia Mahaman Bachir Dodo, Lina Maria Jaime-Tobon, Anne-Gabrielle Harrus, Cécilia Souyris, Jing Wang, Rémy Pujol, Frédéric Venail, Ruben Vidal, Jérôme Bourien, Jean-Luc Puel and Régis Nouvian

is a highly original and deliberate study on the effects of deletion of the ferritin light chain 1 (ftl1) on hearing performance and vulnerability to noise in mice. Specifically, the authors analyzed frequency-specific ABR hearing thresholds and ABR wave I amplitudes, DPOA thresholds and DPOAE amplitudes, endocochlear potential (EP), the cellular and subcellular anatomy of the organ of Corti using scanning EM and transmission EM, and patch-clamp recordings of Ca2+ currents in inner hair cells (IHC). All the methods used are state-of-the art.

The astonishing result of genetically ablating ftl1 is a heterogeneous phenotype in ftl1-/- in that a larger fraction of the ftl1-/- called ftl1-/-LT have normal hearing thresholds at 1 month of age, normal EP, normal microanatomy, normal Ca2+ currents in IHCs whereas a smaller fraction shows strongly elevated hearing thresholds at 1 month, strongly reduced EP, abnormal microanatomy of both IHCs and OHCs, and increased Ca2+ currents in IHCs whereas the other part shows. These effects do not depend on the sex of the animals. The authors found a sex-specific difference in the vulnerability to a mild noise trauma: Increases in ABR thresholds and reductions in ABR wave I amplitudes were smaller in female ftl1+/+ mice compared with female ftl1-/- mice, with the latter behaving approximately like male ftl1+/+ and ftl1-/- mice. In other words, the vulnerability to noise trauma showed a sex-specific difference in ftl1+/+ mice on the C57BL/6J background at the age of 2 – 3 months.

This is a very thorough study with clear hypotheses and an excellent analysis and presentation of the results. The conclusions are straight and justified by the results. The phenotypic heterogeneity in ftl1 KO mice is extremely interesting and will influence the thinking in the field.

Regarding the discussion, genetic considerations should be improved, see paragraph starting at line 362ff, ‘Neither sex, nor (a!) recessive allele contribute to hearing loss in Flt1 KO mice’. It is true that an unknown recessive gene cannot explain the split phenotype in ftl1-/- mice, which is reasoning on the ground of pure Mendelian genetics. Please add few statements on modifier genes/epistasis and on non-linear effects, and that the problem might be solved using whole genome sequencing of ftl1-/-LT versus ftl1-/-HT in the future.

Figures: some thin lines are too thin (e.g. Fig. 7)

The language needs improvement (e.g. excessive use of ‚the‘; hyphens e.g. in ‘time-course’; ‘found out’).

Minor:

Figs. Why do the authors display ABR wave I amplitudes on a logarithmic scale in Fig. 7B,C and Fig. 8A,B whereas in other figures, they use the common linear scale (Fig. 1D-F, Fig. 2E-H, Suppl. Fig. 1G-L, Suppl. Fig. 2D-F?

L. 230 Please rephrase this sentence.
L. 234 Please change '…akin the audiograms…' by ''…akin to the audiograms…'
L. 379 Change to 'Thus, biological sex cannot…'
L. 383 Change to '…function in 1 month- and 5 months-old …
L. 389 and below: I know the 'Mann Whitney u test' and the 'Wilcoxon (rank) test', but I do not know the Mann-Whitney-Wilcoxon's test. What is meant? Please clarify throughout the text.
L. 531f. Change '…in contrast to…' to '…rather than in…'
L. 450 & L. 803: Replace 'gender' by 'sex'
L. 713 change µM to µm

Reviewer #2

(Remarks to the Author)
This study originally describes the importance of ferritin in intracellular iron homeostasis in the cochlea. The authors demonstrate that deletion of the gene encoding a ferritin subunit, its light chain (Ftl), can have various morpho-functional consequences. More specifically, this includes alterations in the stereociliary bundle of inner and outer hair cells, and even cellular death. These phenomena are consistent with the demonstrated deterioration and loss of auditory function. However, intriguingly, the aforementioned alterations occurred in a minor fraction (about 20%) of the KO mice studied. The reason for this, despite the different approaches adopted by the researchers, remains elusive.
In conclusion, this work demonstrates the importance of proper iron homeostasis in ensuring the survival and functionality of cochlear sensory cells. It also suggests the possibility that imperfect iron homeostasis, even when not directly harmful, may make sensory cells more vulnerable to ototoxic factors, such as noise.
This study also lays the foundation for future investigations aimed at clarifying whether a similar vulnerability may be present in relation to other ototoxic agents known to produce reactive oxygen species, such as aminoglycoside antibiotics, whose ototoxicity varies significantly (and unpredictably) from patient to patient.
The statistical analysis is appropriate, as well as the level of details provided.

Please find below my specific comments.

Several correlations have been found between elevated hearing threshold e DPOAEs in KO HT mice, although insufficient to fully explain the complete hearing loss and reduced ABR evoked by high levels of stimulation (line 263-265), reduced EP (in 4 out of 5 KO HT mice; line 271), and altered morphology of IHCs (line 282 – 285) and OHCs (line 294). Furthermore, sex appears to be an important factor in noise vulnerability in Ftl1-/- mice (Line 449-451).
It appears that a critical combination of several factors must be present to cause hearing loss in KO HT mice. Could you comment on this?

The main (unanswered) question is why only about 20% of the KO mice are deaf. The level of Fth expression, which according to Li et al., (2015) can vary and somehow compensate for the lack of Ftl, is taken into account (line 513). However, it should also be mentioned that, for unknown reasons, 50% of these KO mice die before birth (Li et al., 2015). Therefore, it appears that some important information regarding these transgenic mice is missing in the discussion.

Li et al., 2015, studied Flt-/- mice, while your study, while claiming to have used the same transgenic mice as in Li et al., 2015, calls them Flt1-/- mice. Why?

In their paper, Li et al. (2015) reported that 50% Ftl-/- mice, at 2 months of age, exhibited head tilt and a circling behavior in the same direction, which became more prominent and present in more mice as animals aged. My questions are:
1. Have you observed differences in head tilt/circling behavior between LT and HT mice?
2. Is it possible that KO LT mice become HT with age? Five-months old mice (incidentally, this group was not reported in the Methods) were investigated (Supplementary Information 2). Perhaps older animal should also be tested?

Line 51 – "Finally, the Ftl1-/- LT mice were found to be more vulnerable to acoustic exposure, suggesting that the difference between the two phenotypes may partly stem from noxious environment."
However, this appears to be true only for female mice (Line 449-451).

Line 301 and line 309 - There were more damaged hair cells in KO LT mice than in WT mice.
I suggest implementing the relevant conclusion (Line 319) as follows: ".. are likely to undergo a massive alteration in the Ftl1-/- HT mice", and that milder but significant alterations were also discernible in Ftl1-/- LT mice.

Minor
Throughout the manuscript, when referring to the cell membrane voltage or ionic currents, please use the   symbol instead of the - (e.g., 60 mV instead of -60 mV).

Line 91 - We found that in about 20 % of the transgenic mice investigated, deletion of Ftl1 altered hair cells activity and survival, suggesting that iron homeostasis is critical in the auditory function.

Line 152 - Noise at 8-16 kHz, apical HCs were investigated.
What are the characteristic frequencies of the investigated HCs?

Line 230 – … or even no waves could be discerned …

Line 344 – “While the maximum peak of the calcium current was around -100 pA (not mV)

Line 399 – “However, the litter obtained with the aforementioned crossing was populated with 8 Ftl1-/- LT mice and only one Ftl1-/- HT mice”.
This is a very important test. Surprisingly, the fraction of HT mice appears to be lower than the 20% found in heterozygotes matings. Any comment?

Line 405 – “known to produce free radicals such as noise trauma”
Ref?

Line 455 - …impaired calcium signaling…
The properties of the Ca2+ current recorded from KO HT hair cells do not appear to be sufficiently different from those recorded from WT hair cells to justify the above statement. Indeed, some KO HT hair cells in this sub-population appear to exhibit ICa properties similar to those of WT and KO LT hair cells. Furthermore, the 4 mV hyperpolarizing shift (Line 350) would increase Ca2+ channel availability near the cell membrane resting potential. Finally, cell membrane capacitance measurement should be performed to sustain the hypothesis that calcium signaling is impaired.

Line 456 - … appears to stem from a higher susceptibility to acoustic trauma. Yet, (line 427) the noise-induced hearing loss in Ftl1-/- LT mice does not fully recapitulate the phenotype of Ftl1-/- HT mice, given the milder effects on ABR amplitudes and OHC function.
Could you please better clarify this apparent contradiction?

Line 466 – “This finding is similar to the first observation of the Ftl1-/- phenotype, in which the authors observed tilting head and circling behavior among the Ftl-/- (not Ftl1)-/- mice [16]”.

Line 466 - Could you please specify to what extent: did they also observe vestibular dysfunction in about 20% mice?

Line 527 - … only in females given the high susceptibility of wild-type males to noise (REF?)

Line 529 – “…to corroborate the hypothesis that the noise exposure …”

Line 529 – “Further experiments with different protocols are needed to corroborate the noise exposure equally affects male and female Ftl1-/- LT mice.”
In my opinion, demonstrating this would greatly increase the importance of the study.

Line 710 – … stereocilia bundle…

Reviewer #3

(Remarks to the Author)
The article Ferratin light-chain contributes to hair cells function and survival presents a novel auditory phenotype present in mice which lack ferratin light chain. The authors have managed to put together a cohesive analysis of a highly variable phenotype and attempted to find its underlying cause. These findings are of relevance to, not only those in auditory research, but beyond as lack of Ftl1 is likely to have similar impact in other organ systems with high metabolic demand.

In general I found this manuscript without major flaw. The statistical analysis was appropriate and thorough. However, there are a few minor analysis and discussion points which the manuscript would benefit from ahead of publication:

1.I found the argument of the variability observed stemming from acoustic trauma weak and overstated. I would have liked to see ABR data earlier than one-month of age to confirm that this is/isn’t a congenital phenotype (you may already have this as you appear to phenotype your mice taken for electrophysiology?). You also comment on previous findings by Defourny, et al. about litter size being correlated with auditory deficits in the Pejvakin mutants, but do not attempt to perform a similar analysis on your own data.

2. There could be more discussion around the sex differences seen in your acoustic trauma cohort. There is previous evidence to suggest sex differences are observed following noise exposure yet this discussion was lacking.

3. It should be considered that the C57BL/6J background, with the Cdh23-ahl allele, is contributing to your phenotype here by adding additional stress to the auditory system. Would these mice present with a phenotype on a different (non-ahl) background?

Version 1:

Reviewer comments:

Reviewer #1

(Remarks to the Author)
The MS entitled

‚Ferritin light-chain contributes to hair cells function and survival‘

by Chloé P. Petit et al.,

is a highly original and deliberate study on the effects of deletion of the ferritin light chain (ftl) on hearing performance and vulnerability to noise in mice.

Deletion of ftl causes morphological and functional consequences in hair cells, resulting in elevated auditory thresholds of about 20 % of the homozygous mutants yet normal thresholds in the other part of the mutants, independent of the sex of the animals. The ftl-/- high threshold (HT) fraction also showed an increased vulnerability to noise trauma regarding threshold shifts, ABR wave I amplitudes, and DPOAE amplitudes. Electrophysiological properties of inner hair cells were mostly normal, but outer hair cell stereocilia were affected.

The reasons for the segregation of the ftl-/- low threshold (LT) and ftl-/- HT phenotypes are unclear but very interesting. The authors offer explanations for phenotypic segregation. I am convinced that this paper will influence thinking in the field.

The authors describe their work in much detail, providing others with sufficient information to reproduce the work. Statistical analyses are appropriate.

The authors did a great job in the revision. They replied to all my comments and changed the text accordingly.

I only have four minor points:
Line 88: change to "hair cell activity"
Line 88: change to "critical in auditory" or "critical for auditory"
Line 322: replace "in mouse" by "in mice" or by "in the mouse"
Line 492: change to "hydroxyl radicals"

Reviewer #2

(Remarks to the Author)
All my concerns have been adequately addressed in the revised manuscript.

Reviewer #3

(Remarks to the Author)
Thank you for your consideration of my previous comments. I have reviewed the rebuttal letter and manuscript and I am satisfied with the additions and alterations that have been made. The additional data that has been provided in the supplementary complements their arguments and has been appropriately analysed.

Open Access This Peer Review File is licensed under a Creative Commons Attribution 4.0 International License, which permits use, sharing, adaptation, distribution and reproduction in any medium or format, as long as you give appropriate credit to the original author(s) and the source, provide a link to the Creative Commons license, and indicate if changes were made.
In cases where reviewers are anonymous, credit should be given to 'Anonymous Referee' and the source.
The images or other third party material in this Peer Review File are included in the article's Creative Commons license, unless indicated otherwise in a credit line to the material. If material is not included in the article's Creative Commons license and your intended use is not permitted by statutory regulation or exceeds the permitted use, you will need to obtain permission directly from the copyright holder.
To view a copy of this license, visit https://creativecommons.org/licenses/by/4.0/

Reviewers' comments:

Reviewer #1 (Remarks to the Author):

General:

The MS entitled

‚Ferritin light-chain contributes to hair cells function and survival'

by Chloé P. Petit, Sahia Mahaman Bachir Dodo, Lina Maria Jaime-Tobon, Anne-Gabrielle Harrus, Cécilia Souyris, Jing Wang, Rémy Pujol, Frédéric Venail, Ruben Vidal, Jérôme Bourien, Jean-Luc Puel and Régis Nouvian

is a highly original and deliberate study on the effects of deletion of the ferritin light chain 1 (ftl1) on hearing performance and vulnerability to noise in mice. Specifically, the authors analyzed frequency-specific ABR hearing thresholds and ABR wave I amplitudes, DPOA thresholds and DPOAE amplitudes, endocochlear potential (EP), the cellular and subcellular anatomy of the organ of Corti using scanning EM and transmission EM, and patch-clamp recordings of Ca2+ currents in inner hair cells (IHC). All the methods used are state-of-the art.

The astonishing result of genetically ablating ftl1 is a heterogeneous phenotype in ftl1-/- in that a larger fraction of the ftl1-/- called ftl1-/-LT have normal hearing thresholds at 1 month of age, normal EP, normal microanatomy, normal Ca2+ currents in IHCs whereas a smaller fraction shows strongly elevated hearing thresholds at 1 month, strongly reduced EP, abnormal microanatomy of both IHCs and OHCs, and increased Ca2+ currents in IHCs whereas the other part shows. These effects do not depend on the sex of the animals. The authors found a sex-specific difference in the vulnerability to a mild noise trauma: Increases in ABR thresholds and reductions in ABR wave I amplitudes were smaller in female ftl1+/+ mice compared with female ftl1-/- mice, with the latter behaving approximately like male ftl1+/+ and ftl1-/- mice. In other words, the vulnerability to noise trauma showed a sex-specific difference in ftl1+/+ mice on the C57BL/6J background at the age of 2 – 3 months.

This is a very thorough study with clear hypotheses and an excellent analysis and presentation of the results. The conclusions are straight and justified by the results. The phenotypic heterogeneity in ftl1 KO mice is extremely interesting and will influence the thinking in the field.

We thank the reviewer for the advices that improved the discussion and the multiples corrections.

Regarding the discussion, genetic considerations should be improved, see paragraph starting at line 362ff, 'Neither sex, nor (a!) recessive allele contribute to hearing loss in Flt1 KO mice'. It is true that an unknown recessive gene cannot explain the split phenotype in ftl1-/- mice, which is reasoning on the ground of pure Mendelian genetics. Please add few statements on modifier genes/epistasis and on non-linear effects, and that the problem might be solved using whole genome sequencing of ftl1-/-LT versus ftl1-/-HT in the future.

According to the reviewer advice, we changed the discussion and add statements regarding the effect of multiple gene expression:

"Thus, other factors may be responsible for the hearing loss in a proportion of Ftl$^{-/-}$ mice. For instance, the influence of the genetic background on the phenotype cannot be fully discarded (Vénéreau et al., 2017). Indeed, the C57Bl/6J is known to carry the Cdh23-ahl allele (Noben-Trauth et al., 2003), which may impose additional stress on the auditory system. Further experiments, which consist to study the Ftl1 deletion in another strain or to carry-out whole genome sequencing, are required to test this hypothesis. In addition, loss of Ftl leads to embryonic lethality in approximately 50% of the expected Ftl$^{-/-}$ embryos (Li et al., 2015). The authors raised the hypothesis that the light-chain plays a pivotal role in mouse embryogenesis (González-Morales, et al., 2015) and that ferritin heavy chain may rescue, albeit not completely, the loss of the light chain (Li et al., 2015). A similar scheme may apply to the cochlea."

Figures: some thin lines are too thin (e.g. Fig. 7)

Corrected

The language needs improvement (e.g. excessive use of ‚the'; hyphens e.g. in 'time-course'; 'found out').

Corrected

Minor:

Figs. Why do the authors display ABR wave I amplitudes on a logarithmic scale in Fig. 7B,C and Fig. 8A,B whereas in other figures, they use the common linear scale (Fig. 1D-F, Fig. 2E-H, Suppl. Fig. 1G-L, Suppl. Fig. 2D-F?

In Fig 7, we compared the amplitude of the ABR across different intensity level of stimulation, from 10 to 80 dB SPL. The log scale allows to appreciate the changes in the ABR amplitude for low-level of stimulation. In the other figures, ABR are evoked by a tone burst at 80dB SPL.

L. 230 Please rephrase this sentence.

"*While audiograms that looked similar to the wild-type mice were recorded in most of the Ftl1$^{-/-}$ mice, auditory thresholds that could reach 100 dB SPL were measured in a fraction of homozygous mice*" has been changed to "*While audiograms that looked similar to the wild-type mice were recorded in most of the Ftl$^{-/-}$ mice, <u>elevated auditory thresholds were measured in a fraction of homozygous mice</u>.* "

L. 234 Please change '…akin the audiograms…' by ''…akin to the audiograms…'

Done

L. 379 Change to 'Thus, biological sex cannot…'

Done

L. 383 Change to '…function in 1 month- and 5 months-old …

Done

L. 389 and below: I know the 'Mann Whitney u test' and the 'Wilcoxon (rank) test', but I do not know the Mann-Whitney-Wilcoxon's test. What is meant? Please clarify throughout the text.

The referee is right. We corrected the name of the test according to the comment of the referee. We made a mistake (basically, we took the name of the toolbox from Igor, which combines the 3 names).

L. 531f. Change '…in contrast to…' to '…rather than in…'

Done

L. 450 & L. 803: Replace 'gender' by 'sex'

Done

L. 713 change µM to µm

Done

Reviewer #2 (Remarks to the Author):

This study originally describes the importance of ferritin in intracellular iron homeostasis in the cochlea. The authors demonstrate that deletion of the gene encoding a ferritin subunit, its light chain (Ftl), can have various morpho-functional consequences. More specifically, this includes alterations in the stereociliary bundle of inner and outer hair cells, and even cellular death. These phenomena are consistent with the demonstrated deterioration and loss of auditory function. However, intriguingly, the aforementioned alterations occurred in a minor fraction (about 20%) of the KO mice studied. The reason for this, despite the different approaches adopted by the researchers, remains elusive.
In conclusion, this work demonstrates the importance of proper iron homeostasis in ensuring the survival and functionality of cochlear sensory cells. It also suggests the possibility that imperfect iron homeostasis, even when not directly harmful, may make sensory cells more vulnerable to ototoxic factors, such as noise.
This study also lays the foundation for future investigations aimed at clarifying whether a similar vulnerability may be present in relation to other ototoxic agents known to produce reactive oxygen species, such as aminoglycoside antibiotics, whose ototoxicity varies significantly (and unpredictably) from patient to patient.
The statistical analysis is appropriate, as well as the level of details provided.

We thank the reviewer for the advice that help us to add further hypotheses underlying the mechanisms for the two phenotypes in the Ftl knock-out colony.

Please find below my specific comments.

Several correlations have been found between elevated hearing threshold e DPOAEs in KO HT mice, although insufficient to fully explain the complete hearing loss and reduced ABR evoked by high levels of stimulation (line 263-265), reduced EP (in 4 out of 5 KO HT mice; line 271), and altered morphology of IHCs (line 282 – 285) and OHCs (line 294). Furthermore, sex appears to be an important factor in noise vulnerability in Ftl1-/- mice (Line 449-451). It appears that a critical combination of several factors must be present to cause hearing loss in KO HT mice. Could you comment on this?

We agree with the reviewer that several factors contribute to the hearing loss in the *Ftl1*$^{-/-}$ *HT* mice, as stated in the discussion: "*Taken together, both defective IHCs and OHCs with the EP reduction contribute to the hearing loss in the Ftl1*$^{-/-}$ *HT mice*." It is however difficult to determine the key factor that leads to the multiple alterations, but the ubiquitous expression of ferritin may explain the alterations in several cell types (stated in the discussion). We would like nevertheless point that the sex-dimorphism regarding the noise vulnerability has been previously reported and involved OHC and ribbon synapses protection through estradiol signaling (Meltser et al., 2008; Shuster et al., 2021). In addition, following the advice of the reviewers, we have toned down the hypothesis that noise exposure within the animal facility may be responsible of the divergent phenotype. Indeed, noise within the animal facility would affect all the individuals in the litter rather than in a proportion of mice. We have also observed a fraction of *Ftl*$^{-/-}$ *HT* mice right after the onset of hearing, corresponding to a period in which the cochlea has not yet reached full maturity, and should be still insensitive to noise trauma (Bock and Saunders, 1977).

The main (unanswered) question is why only about 20% of the KO mice are deaf. The level of Fth expression, which according to Li et al., (2015) can vary and somehow compensate for the lack of Ftl, is taken into account (line 513). However, it should also be mentioned that, for unknown reasons, 50% of these KO mice die before birth (Li et al., 2015). Therefore, it appears that some important information regarding these transgenic mice is missing in the discussion.

According to the reviewer advice, we changed the discussion and add statements regarding the effect birth rate in the FTL colony. *"In addition, loss of Ftl leads to embryonic lethality in approximately 50% of the expected Ftl$^{-/-}$ embryos (Li et al., 2015). The authors raised the hypothesis that the light-chain plays a pivotal role in mouse embryogenesis (González-Morales, et al., 2015) and that ferritin heavy chain may rescue, albeit not completely, the loss of the light chain (Li et al., 2015). A similar scheme may apply to the cochlea."*

Li et al., 2015, studied Flt-/- mice, while your study, while claiming to have used the same transgenic mice as in Li et al., 2015, calls them Flt1-/- mice. Why?

We used the same mice. Ferritin light chain can be designed by both aliases and Ftl1 is the name according to the Mouse Genome Informatics database. However, to avoid any confusion, we modified the MS and figures with Ftl instead of Ftl1.

In their paper, Li et al. (2015) reported that 50% Ftl-/- mice, at 2 months of age, exhibited head tilt and a circling behavior in the same direction, which became more prominent and present in more mice as animals aged. My questions are:
1. Have you observed differences in head tilt/circling behavior between LT and HT mice?

We also observed head tilt and circling behavior in approximatively 40% of the *Ftl$^{-/-}$ HT* population (estimation made by eyes). Given we did not perform quantitative measurements regarding this behavior, we did not mention this in the MS.

2. Is it possible that KO LT mice become HT with age? Five-months old mice (incidentally, this group was not reported in the Methods) were investigated (Supplementary Information 2). Perhaps older animal should also be tested?
The reviewer is right, but given the genetic background of the mice, C57BL/6J, known to develop progressive hearing loss over time, we should repeat these experiments using alternative genetic background. We add now the group in the methods.

Line 51 – "Finally, the Ftl1-/- LT mice were found to be more vulnerable to acoustic exposure, suggesting that the difference between the two phenotypes may partly stem from noxious environment." However, this appears to be true only for female mice (Line 449-451).
We took into account the comment of the reviewer, by adding the following paragraph in the discussion:

The sex-dimorphism regarding the noise vulnerability has been previously reported and involved OHC and ribbon synapses protection through estradiol signaling (Meltser et al., 2008; Shuster et al., 2021). Because our acoustic exposure paradigm makes the noise consequence indistinguishable between wild-type and Ftl$^{-/-}$ male mice, further experiments with different protocols are needed to corroborate the hypothesis that the noise exposure equally affects male and female Ftl$^{-/-}$ LT mice (Kujawa and Liberman, 2006)"

As state below, we have toned down the hypothesis that noise exposure within the animal facility may be responsible of the divergent *phenotype*. *"Finally, a fraction of Ftl$^{-/-}$ HT mice is observed right after the onset of hearing, corresponding to a period in which the cochlea did not reach full maturity, and hence is still insensitive to noise trauma (Bock and Saunders, 1977). For instance, the influence of the genetic background on the phenotype cannot be fully discarded (Vénéreau et al., 2017). Indeed, the C57BL/6J is known to carry the Cdh23-ahl allele (Noben-Trauth et al., 2003), which may impose additional stress on the auditory system. Further experiments, which consist to study the Ftl deletion in another strain or to carry-out whole genome sequencing, are required to test this hypothesis."*

Line 301 and line 309 - There were more damaged hair cells in KO LT mice than in WT mice.
I suggest implementing the relevant conclusion (Line 319) as follows: ".. are likely to undergo a massive alteration in the Ftl1-/- HT mice", and that milder but significant alterations were also discernible in Ftl1-/- LT mice.
We corrected the MS according to the reviewer's advice.

Minor
Throughout the manuscript, when referring to the cell membrane voltage or ionic currents, please use the – symbol instead of the - (e.g., –60 mV instead of -60 mV).
Done

Line 91 - We found that in about 20 % of the transgenic mice investigated, deletion of Ftl1 altered hair cells activity and survival, suggesting that iron homeostasis is critical in the auditory function.
Done

Line 152 - Noise at 8-16 kHz, apical HCs were investigated.
What are the characteristic frequencies of the investigated HCs?
At 85 dB SPL, the noise exposure between 8-16 kHz lead to a threshold elevation spanning approximately half to one octave above the exposure frequency band, consistent with established noise trauma paradigm.

Line 230 – … or even no waves could be discerned …

Corrected according to the reviewer 3

Line 344 – "While the maximum peak of the calcium current was around -100 pA (not mV)

Done

Line 399 – "However, the litter obtained with the aforementioned crossing was populated with 8 Ftl1-/- LT mice and only one Ftl1-/- HT mice".
This is a very important test. Surprisingly, the fraction of HT mice appears to be lower than the 20% found in heterozygotes matings. Any comment?

In the offspring from Ftl$^{-/-}$ x Ftl$^{-/-}$, the proportion of Ftl1$^{-/-}$ HT mice is highly variable, ranging from 0 to 50% across litters. The reasons for this variability remain unclear but the fluctuations in the *Ftl$^{-/-}$ HT* number may reflect heterogeneous developmental outcomes, including embryonic loss. Although we have observed 23 % of *Ftl$^{-/-}$ HT* in the first set of experiments, this proportion was 18% across the entire study. This is now stated in the MS.

Line 405 – "known to produce free radicals such as noise trauma" Ref?

Ref included (Ohlemiller et al., 1999)

Line 455 - …impaired calcium signaling…
The properties of the Ca2+ current recorded from KO HT hair cells do not appear to be sufficiently different from those recorded from WT hair cells to justify the above statement. Indeed, some KO HT hair cells in this sub-population appear to exhibit ICa properties similar to those of WT and KO LT hair cells. Furthermore, the 4 mV hyperpolarizing shift (Line 350) would increase Ca2+ channel availability near the cell membrane resting potential. Finally, cell membrane capacitance measurement should be performed to sustain the hypothesis that calcium signaling is impaired.
We definitively agree with the reviewer so we changed this statement to calcium current instead of signaling.

Line 456 - … appears to stem from a higher susceptibility to acoustic trauma. Yet, (line 427) the noise-induced hearing loss in Ftl1-/- LT mice does not fully recapitulate the phenotype of Ftl1-/- HT mice, given the milder effects on ABR amplitudes and OHC function.
Could you please better clarify this apparent contradiction?

Using our acoustic exposure (8-16 kHz, 85 dB SPL), the reduction in ABR and DPOAEs did not reach those of the *Ftl*$^{-/-}$ *HT* mice. This is why we emphasized a milder effect. We re-wrote this sentence to make it clear. *"However, the noise-induced hearing loss in Ftl*$^{-/-}$ *LT mice does not fully recapitulate the phenotype of Ftl*$^{-/-}$ *HT mice, as the reductions in ABR and DPOAEs did not reach those observed in Ftl*$^{-/-}$ *HT mice."*

Line 466 – "This finding is similar to the first observation of the Ftl1-/- phenotype, in which the authors observed tilting head and circling behavior among the Ftl-/- (not Ftl1)-/- mice [16]".
Line 466 - Could you please specify to what extent: did they also observe vestibular dysfunction in about 20% mice?

The authors observed tilting head and circling behavior in approximately 50% of the Ftl$^{-/-}$ mice @ approximatively 2 months of age. We also observed head tilt and circling behavior in approximatively 40% of the *Ftl*$^{-/-}$ *HT* population (estimation made by eyes). We rewrote the sentence to make it clear. *"This finding is consistent with the first observation of the Ftl*$^{-/-}$ *phenotype, in which the authors observed tilting head and circling behavior among the Ftl*$^{-/-}$ *mice, indicative of a potential alteration in the inner ear."*

Line 527 - … only in females given the high susceptibility of wild-type males to noise (REF?)

References are indicated now (Milon et al. 2018; Fernandez et al., 2020; Schrode et al., 2022).

Line 529 – "…to corroborate the hypothesis that the noise exposure …"

Done

Line 529 – "Further experiments with different protocols are needed to corroborate the noise exposure equally affects male and female Ftl1-/- LT mice."
In my opinion, demonstrating this would greatly increase the importance of the study.

We definitively agree with the reviewer, but finding the right noise-exposure paradigm is time- and resources demanding, which will be difficult to carry-out in a reasonable time-window.

Line 710 – … stereocilia bundle…
Done

Reviewer #3 (Remarks to the Author):

The article Ferritin light-chain contributes to hair cells function and survival presents a novel auditory phenotype present in mice which lack ferratin light chain. The authors have managed to put together a cohesive analysis of a highly variable phenotype and attempted to find its underlying cause. These findings are of relevance to, not only those in auditory research, but beyond as lack of Ftl1 is likely to have similar impact in other organ systems with high metabolic demand.

In general I found this manuscript without major flaw. The statistical analysis was appropriate and thorough. However, there are a few minor analysis and discussion points which the manuscript would benefit from ahead of publication:
We thank the reviewer for the advices that improved the discussion and the multiples corrections.

1.I found the argument of the variability observed stemming from acoustic trauma weak and overstated. I would have liked to see ABR data earlier than one-month of age to confirm that this is/isn't a congenital phenotype (you may already have this as you appear to phenotype your mice taken for electrophysiology?). You also comment on previous findings by Defourny, et al. about litter size being correlated with auditory deficits in the Pejvakin mutants, but do not attempt to perform a similar analysis on your own data.

When we recorded ABR right after the onset of hearing (P15-P16), mice from the Ftl1 KO colony displayed heterogeneous auditory phenotypes: some exhibited normal ABR thresholds and amplitudes, whereas others showed elevated thresholds and reduced amplitudes (now shown in Supplementary Fig. 1A-C). To minimize prolonged anesthesia, which led to increased mortality, we used DPOAEs to monitor cochlear function, as this technique is faster to perform than ABR recordings. Consistent with the ABR findings, a fraction of mice exhibited reduced DPOAE amplitudes (now shown in Supplementary Fig. 1D-I). In most of the P15 mice, the sensory epithelia were properly organized with decent stereociliary bundles (Supplementary Fig. 1J-R). Out of 5 cochlea from 3 $Ftl^{-/-}$ HT mice, only one contained hair cells with splayed and missing stereocilia (approximatively 20% and 15 % of IHCs and OHCs with splayed stereocilia, respectively, and 10 % of OHCs with missing stereocilia, SI 1S-T). These data suggest that i) the functional loss most likely precedes the morphological damages and ii) acoustic trauma is not a satisfactory explanation for the phenotype variability as hearing loss readily occurs at the onset of hearing, a period in which the cochlea has not yet reached full maturity and is still relatively insensitive to noise exposure (Bock and Saunders, 1977).

Moreover, environmental noise within the animal facility would be expected to affect all individuals within a litter rather than only a subset of mice. Thus, the reviewer's comment convinced us to tone down the noise trauma hypothesis in the MS. Regarding the correlation between litter size and number of animals with hearing loss, a major limitation in our study is that breeding and hence the litters were not housed in a dedicated room within the animal facility. Several other colonies and thus multiples cages, that can fluctuate over times, are hosted in the same room and a decent correlation would merit to get the background noise. Thus, we do not feel comfortable to make a similar correlation as Defourny et al..

Instead, as suggested by Reviewer 1, we now propose the alternative hypothesis that loss of the ferritin light chain may be essential at the onset of hearing. In this scenario, partial compensation by the ferritin heavy chain could occur, potentially accounting for the variable penetrance of the phenotype.

2. There could be more discussion around the sex differences seen in your acoustic trauma cohort. There is previous evidence to suggest sex differences are observed following noise exposure yet this discussion was lacking.

According to the reviewer, we now add more reference on the sex difference following acoustic exposure.*" First, the noise paradigm we used in our study provokes threshold shift only in female given the larger susceptibility of wild-type male to noise (Milon et al., 2018; Schrode et al., 2022). The sex-dimorphism regarding the noise vulnerability has been previously reported and involved OHC and ribbon synapses protection through estradiol signaling (Meltser et al., 2008; Shuster et al., 2021)."*

3. It should be considered that the C57BL/6J background, with the Cdh23-ahl allele, is contributing to your phenotype here by adding additional stress to the auditory system. Would these mice present with a phenotype on a different (non-ahl) background?

We agree with the referee that the Cdh23-ahl allele may be a genetic confounder of the phenotype (Noben-Trauth et al., 2003). By (bad) habit, we kept on the C57BL/6J line, arguing that hearing loss progressively starts at 3 months of age (Hequembourg and Liberman, 2001). Therefore, the possibility that the genetic background influence the phenotype cannot be fully discarded and is now raised in the MS.

"Thus, other factors may be responsible for the hearing loss in a proportion of $Ftl^{-/-}$ mice. For instance, the influence of the genetic background on the phenotype cannot be fully discarded (Vénéreau et al., 2017). Indeed, the C57BL/6J is known to carry the Cdh23-ahl allele (Noben-Trauth et al., 2003), which may impose additional stress on the auditory system. Further experiments, which consist to study the Ftl deletion in another strain or to carry-out whole genome sequencing, are required to test this hypothesis. In addition, loss of Ftl leads to embryonic lethality in approximately 50% of the expected $Ftl^{-/-}$ embryos (Li et al., 2015). The authors raised the hypothesis that the light-chain plays a pivotal role in mouse embryogenesis (González-Morales, et al., 2015) and that ferritin heavy chain may rescue, albeit not completely, the loss of the light chain (Li et al., 2015). A similar scheme may apply to the cochlea."
